# The *Rosa chinensis* cv. Viridiflora Phyllody Phenotype Is Associated with Misexpression of Flower Organ Identity Genes

**DOI:** 10.3389/fpls.2016.00996

**Published:** 2016-07-12

**Authors:** Huijun Yan, Hao Zhang, Qigang Wang, Hongying Jian, Xianqin Qiu, Sylvie Baudino, Jeremy Just, Olivier Raymond, Lianfeng Gu, Jihua Wang, Mohammed Bendahmane, Kaixue Tang

**Affiliations:** ^1^Flower Research Institute of Yunnan Academy of Agricultural SciencesKunming, China; ^2^Université de Lyon, UJM-Saint-Etienne, CNRS, BVpam FRE 3727Saint-Etienne, France; ^3^Laboratoire de Reproduction et Développement des Plantes, Université de Lyon, ENS de Lyon, UCB Lyon 1, CNRS, INRALyon, France; ^4^Haixia Institute of Science and Technology, Fujian Agriculture and Forestry UniversityFuzhou, China

**Keywords:** rose, Viridiflora, phyllody, transcriptome analysis, ABC flower organ’s identity genes, *RcSOC1*

## Abstract

Phyllody is a flower abnormality in which leaf-like structures replace flower organs in all whorls. Here, we investigated the origin and the molecular mechanism of phyllody phenotype in *Rosa chinensis* cv. Viridiflora, an ancient naturally occurring Chinese mutant cultivar. Reciprocal grafting experiments and microscopy analyses, demonstrated that the phyllody phenotype in Viridiflora is not associated with phytoplasmas infection. Transcriptome comparisons by the mean of RNA-Seq identified 672 up-regulated and 666 down-regulated genes in Viridiflora compared to its closely related genotype *R. chinensis* cv. Old Blush. A fraction of these genes are putative homologs of genes known to be involved in flower initiation and development. We show that in flower whorl 2 of Viridiflora, a down-regulation of the floral organ identity genes *RcPISTILLATA (RcPI), RcAPETALA3 (RcAP3)* and *RcSEPALLATA3 (RcSEP3)*, together with an up-regulation of the putative homolog of the gene *SUPPRESSOR of OVEREXPRESSION of CONSTANS1* (*RcSOC1)* are likely at the origin of the loss of petal identity and leaf-like structures formation. In whorl 3 of Viridiflora, ectopic expression of *RcAPETALA2* (*RcAP2*) along with the down regulation of *RcPI*, *RcAP3*, and *RcSEP3* is associated with loss of stamens identity and leaf-like structures formation. In whorl 4, the ectopic expression of *RcAP2* associated with a down-regulation of *RcSEP3* and of the C-class gene *RcAGAMOUS* correlate with loss of pistil identity. The latter also suggested the antagonist effect between the A and C class genes in the rose. Together, these data suggest that modified expression of the ABCE flower organ identity genes is associated with the phyllody phenotype in the rose Viridiflora and that these genes are important for normal flower organs development.

## Introduction

**FIGURE 1:**
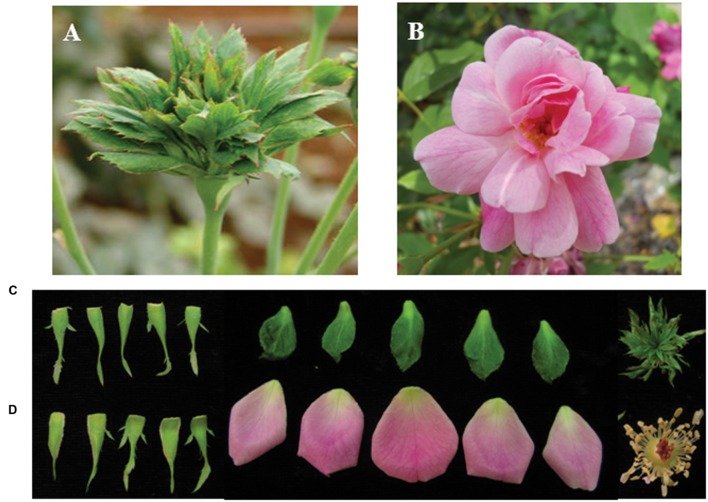
**Flower phenotype of *Rosa chinensis* cv. Viridiflora **(A,C)** and Old Blush **(B,D)**.** **(C,D)** Dissected flower organs of Viridiflora and Old Blush, respectively.

Roses have been cultivated by humans since antiquity, as early as 3 000 B.C. in China. Today there exist more than 35 000 rose cultivars. These modern rose cultivars were established from less than 10 rose species, including Chinese species (Wylie, 1954; Liu, 1964). Chinese old roses have high ornamental, cultural, economic values, and represent an important rose germplasms resource (Zhang and Zhu, 2006; Yan et al., 2014). Among old Chinese rose species, the recurrent blooming *Rosa chinensis* was used in many breeding programs to select for the most advantageous traits such as recurrent blooming, scent and resistance to pathogens (Martin et al., 2001; Ku and Robertson, 2003; Bendahmane et al., 2013). *R. chinensis* cv. Viridiflora (hereafter Viridiflora) is a rose cultivar, commonly known as the ‘green rose’ (Chmelmitsky et al., 2002), in which petals, stamens and pistils are converted into leaf-like organs (**Figures 1A,C**). This phenomenon is also known as phyllody, a phenotype that has been described in many plant species (Meyer, 1966; Mor and Zieslin, 1992). Viridiflora is a spontaneous mutant of *R. chinensis* Jaquin (Krussman, 1981). This stable mutant has been maintained for over 200 years in China, Europe and America, and is widely used for ornamental horticulture.

The prevailing ABCE genetic model of floral organ identity determination, with the combinatorial activity of four classes of homeotic genes (A, B, C, and E), has been widely characterized in many flowering species. Most of the ABCE genes encode MADS-box transcription factors (Coen and Meyerowitz, 1991; Smaczniak et al., 2012). In *Arabidopsis*, the class A-class genes *APETALA1* (*AP1*) and *APETALA2* (*AP2*) specify sepal organ identity and development; A-class genes and the B-class genes *APETALA3* (*AP3*) and *PISTILLATA* (*PI*) together determine petal organ identity and development; B-class genes together with the C-class gene *AGAMOUS* (*AG*) are required for stamen formation and the C-class gene *AG* is required for carpel formation (Weigel and Meyerowitz, 1994). The E-class genes *SEPALLATA* (*SEP1*, *SEP2*, *SEP3*, *SEP4*) interact with A, B, and C class genes and are involved in specifying floral organs in all flower whorls. The ABCE model is relatively conserved in the flowering plants with few variations (Heijmans et al., 2012; Smaczniak et al., 2012; Wellmer et al., 2014).

In *Rosa* sp., homologs of A-class (*RhAP1, RhAP2*), B-class (*RhAP3*, *RhPI*), C-class (*RhAG*) and E-class genes have been reported (Dubois et al., 2012; Bendahmane et al., 2013). Expression analysis and overexpression experiments of these rose MADS-encoding cDNAs in *Arabidopsis* suggested their role in flower organ identity determination in the rose (Kitahara and Matsumoto, 2000; Kitahara et al., 2004; Hibino et al., 2006; Dubois et al., 2010, 2011, 2012; Ma et al., 2015).

In *Arabidopsis*, loss of function of A, B, or C genes lead to homeotic conversion of floral organs and floral aberrations that are different from the phyllody-like phenotype (Coen and Meyerowitz, 1991). In the rose it was demonstrated that misexpression or down-regulation of the *RhAG* in whorl 3 was associated with homeotic conversion of stamens to petals and double flower formation (Dubois et al., 2010). In *Arabidopsis*, the triple *SEP1/2/3* mutant produces flowers in which all organs develop as sepals (Pelaz et al., 2000). It was also reported in many plants that, infection by phytoplasmas was also reported to lead to phyllody (McCoy et al., 1989; Szyndel, 2003; Hogenhout et al., 2008). In *Arabidopsis*, it was recently shown that the phytoplasma-secreted protein PHYL1 is involved in the targeted protein degradation of the organ identity proteins SEP3 and AP1, which in turn lead to the transformation of flower organs into leaf-like structures (Maejima et al., 2014, 2015).

In this study, we addressed the cause of phyllody phenotype in the rose Viridiflora. We show that phyllody phenotype in Viridiflora is not caused by Mycoplasma like organism (MLO) infection. To identify the molecular basis of the malformed flowers in Viridiflora, we used a transcriptomic approach to compare gene expression in flowers of *R. chinensis* cv. Old Blush (hereafter Old Blush) and Viridiflora. Our study identified that the green flower phenotype (phyllody) in *R. chinensis* cv. Viridiflora is associated with misexpression of the putative homologs of the flowering integrator *RcSOC1* and of ABCE flower organ identity genes *RcAP1*, *RcAP2*, *RcPI*, *RcAG*, and *RcSEP3*.

## Materials and Methods

### Plant Materials and Grafting Experiment

*Rosa chinensis* cv. Old Blush (**Figures 1B,D**) and its variant *R. chinensis* cv. Viridiflora, in which petals, stamens, and pistils are converted to leaf-like organs (**Figures 1A,C**), were field-grown in Flower Research Institute of Yunnan, Academy of Agricultural Science. A part from the phyllody phenotype, no other phenotypic differences were observed between Old Blush and Viridiflora (**Figures 1A,C**). Flower buds at stage 8–10 mm were collected and then immediately frozen in liquid nitrogen until RNA extraction.

**FIGURE 2:**
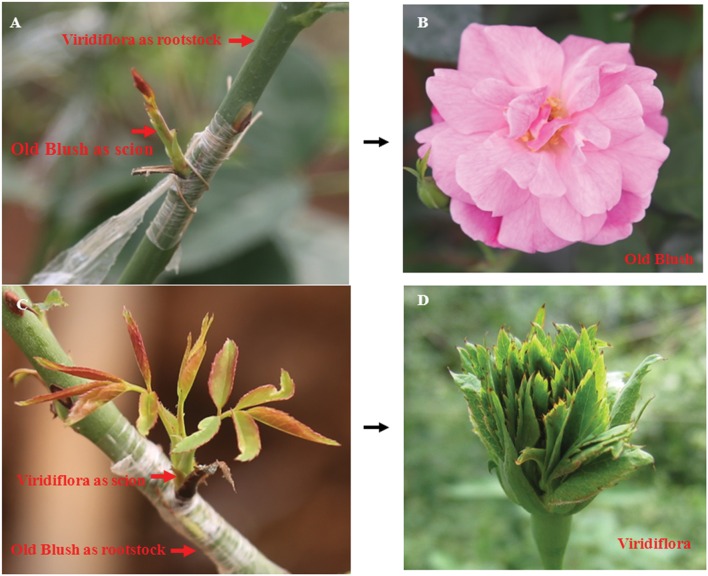
**Grafting does not lead to modified floral phenotype of Viridiflora and Old Blush.** **(A,B)** Shoots of Old Blush were grafted on Viridiflora used as rootstock. Mature flower of Old Blush with Viridiflora as rootstock show wild-type Old Blush flower phenotype **(B)**; **(C,D)** Shoots of Viridiflora were grafted on Old Blush used as rootstock. Mature flower of Viridiflora with Old Blush as rootstock show phyllody phenotype **(D)**.

Grafting experiments were performed as previously described (Ohkawa, 1980). Old Blush young shoots, used as scion, were grafted on Viridiflora plants used as rootstock. As control Viridiflora young shoots, used as scion, were grafted on Old Blush plants used as rootstock (**Figures 2A,C**). Twenty shoot grafting experiments were performed for each.

### Microscopy and PCR Experiment to Detect Mycoplasma

To detect mycoplasma, samples of about 2 mm^2^ were cut from young flower buds, then crude sap extracts were prepared from these tissues in 0.01 M potassium phosphate pH 7.0 buffer and then applied to 4% Formvar-coated, carbon-stabilized copper grids (300 mesh). Grids were then stained with 2% uranyl acetate for 10 s, rinsed with 0.01 M potassium phosphate pH 7.0 buffer and water (Yin et al., 2014). Grids were dried and examined on a JEM-100CX II transmission electron microscope (JOEL, Ltd, Tokyo, Japan).

Total DNA was extracted from young flower buds using Plant DNA Isolation Reagent (TakaRa, China) and then used to PCR amplify MLO DNA using the MLO specific primers R16mF/R16mR (Lee et al., 1993; **Supplementary Table S1**). A construct harboring MLO DNA was used as positive control.

### Library Construction and RNA-Sequencing

Total RNA was extracted using TRIzol reagent and contaminating DNA was then removed by treatment with RNase-free DNase. NanoDrop and Agilent Technologies 2100 Bioanalyzer were used to quantify the total RNA before RNA-Seq library construction.

Transcriptome libraries of Viridiflora and Old Blush were constructed using Illumina TruSeq RNA sample preparation Kit V2 following the manufacturer’s instructions. In brief, total RNA was purified using Magnetic Oligo (dT) beads. Then, purified RNA was sheared to approximately 330 nucleotides fragments and primed for cDNA synthesis using random primers. Subsequently, the fragments were ligated to sequencing adapters. Using agarose gel electrophoresis, the suitable fragments with 400–500 nt sizes were selected as templates for PCR amplification and the final PCR products were sequenced using Illumina HiSeq 2500 system as 125 nt, paired-end Illumina reads.

### Transcriptome Assembly and Annotation

High-quality transcript sequences were obtained by *de novo* transcriptome assembly of the RNA-seq reads, using Trinity software (version: r20140413p1; Langmead and Salzberg, 2012) with default parameters. Open reading frame (ORF) identification was identified using Transdecoder (Grabherr et al., 2011) with default parameters. Homology sequences search was performed against the UniProt databases by ncbi-blast-2.2.27+ with an E-value of 10^-6^. The GO terms were assigned to each assembled sequences based on the UniProt databases. The all information of transcriptome data is available in the database^1^

### Gene Expression Analysis and DEG Identification

The read counts for each transcript were calculated after aligning the RNA-Seq reads on the assembled transcriptome using Bowtie2 2.1.0 (Langmead and Salzberg, 2012). To accurately measure gene expression level, we only retained pairs of reads having both ends matching on the same transcript. The expression level of each transcript was normalized as the fragments per kilobase of exon per million fragments mapped (FPKM), which is analogous to single-read RPKM for single reads (Mortazavi et al., 2008). The *P-*value were calculated according to the statistical R package DEGseq (Wang et al., 2010) using MA-plot-based method with random sampling model. Then, the differentially expressed genes were identified using fold-change >3 and a *P* < 0.001 as the threshold.

### Validation of Differential Expressed Genes

The sequences of the genes used for qRT-PCR validation were provided (details listed in **Supplementary Data Sheet S1**). Gene-specific primers (**Supplementary Table S1**) were designed using Primer3^2^ Total RNAs were extracted from leaves and from sepals, petals, stamens, and pistils at 8–10 mm flower buds development stage using TRIzol RNA purification kit (TaKaRa, China). One microgram of total RNA was used in reverse transcription in a total reaction volume of 20 μL in the presence of 6-mer random primers and an oligo primer according to the protocol provided by manufacturer TaKaRa (Yan et al., 2011). The standard curve for each gene was obtained by real-time PCR with five dilutions of cDNA. The reactions were performed in 20 μL volumes each containing 10 μL 2× SYBR Green Mastermix (TaKaRa), 300 nM of each primer and 2 μL of 10-fold diluted cDNA template (Yan et al., 2014). The PCR reactions were run in a Bio-Rad Sequence Detection System. Three biological replicates were performed for each analysis. *RhGAPDH* (AB370120) was used as control. Quantification of gene relative expression in different organs was performed using the delta-delta Ct method as described by Livak and Schmittgen (2001). All data were expressed as the mean ± standard deviation (SD) after normalization.

## Results

### Phyllody Phenotype of *R. chinensis* cv. Viridiflora Is Not Associated with Phytoplasma

Viridiflora refers to a floral aberration in which petals, stamens, and pistils are converted to leaf-like organs (**Figures 1A,C**) a phenotype that resembles phytoplasmas infection-induced phyllody phenotype. Phytoplasmas are non-cultivable microorganisms transmitted by contact with infected plants. The grafting mechanical contact between the rootstock and the scion enables the spread of this disease and this approach is used generally to detect phytoplasmas (Golino et al., 1989; Aldaghi et al., 2007; Goldschmidt, 2014). To address if Viridiflora was associated with mycoplasma infection, we used grafting approach in which Viridiflora was used as rootstock and healthy Old Blush was used as scion (**Figure 2A**) and *vice versa*, used as control (**Figure 2C**). Twenty bud grafting experiments were performed for each. These data showed that all Old Blush buds with Viridiflora as rootstock bloomed normally and flower organs phenotype was identical to non-grafted Old Blush control plants (**Figure 2B**). At the same time, all Viridiflora flowers with Old Blush as rootstock exhibited phyllody phenotype similar to the none grafted Viridiflora control plants (**Figure 2D**). It should be noted that flowers of Old Bush used as rootstock, showed no phyllody phenotype. These data suggest that phyllody phenotype in Viridiflora is not a result of phytoplasma infection. In agreement with these data, transmission electron microscopy or PCR experiments identified no trace of phytoplasma or phytoplasma DNA respectively, in young buds of Viridiflora and Old Blush (**Supplementary Figures S1** and **S2**). Taken together these data strongly suggest that the flower mutant phenotype of Viridiflora was not caused by phytoplasma infection.

### Flower Buds Transcriptome Comparison in Viridiflora and Old Blush

**FIGURE 3:**
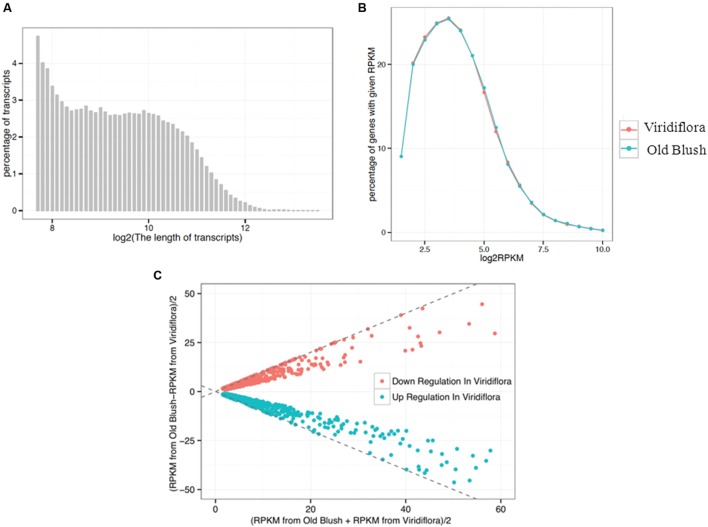
**Sequence length distribution of assembled transcripts **(A)**, plotting distribution of log-transformed FPKM (Fragments per kilobase of exon per million fragments mapped) values **(B)** and MA-plot for differential expressed genes **(C)**.** **(A)** Displays the distribution of transcripts length range. **(B)** Shows the percentage of genes with a given FPKM value. **(C)** Shows the differential expressed genes. The X-axis indicates the value of [FPKM from Old Blush + FPKM from Viridiflora]/2, and the Y-axis indicates the value of [FPKM from Old Blush – FPKM from Viridiflora]/2.

To investigate further the molecular basis of the phyllody phenotype, we compared the transcriptome in flowers of Viridiflora and Old Blush. Total RNA was prepared from flower buds at 8–10 mm development stage and then used to build two libraries for high-throughput sequencing. A total of 40 and 44 million reads were generated from Viridiflora and Old Blush samples, respectively. The transcriptome assembly sequences of *R. chinensis* cv. Viridiflora has been deposited at the Database of Transcriptome Shotgun Assembly (TSA) at DDBJ/EMBL/GenBank under the accession GETJ00000000. The transcriptome data are available in the database^1^. In total, five gigabase for each library were produced. Paired-end reads were assembled into transcript sequences and yielded 68,565 unisequences with an average length of 887 bp (**Figure 3A**). Approximately 91% of the reads from Viridiflora and Old Blush could be successfully aligned to the assembled transcripts, used as reference sequences (**Table 1**). In total, over 32,224,620 and 34,200,388 reads were mapped back in pairs with the unique location for Viridiflora and Old Blush libraries, respectively (**Table 1**).

**Table 1:** Summary of Illumina sequencing and assembly for two RNA-Seq libraries.

Sample	Reads (PE)	Length	Total bases	GC%	Mapping (ratio)	Unique (ratio)	FPKM > 3
Viridiflora	20,640,280 × 2	125,125	5,160,070,000	46,46	37,697,457(91%)	16,112,310 × 2(78%)	20,017
Old Blush	22,052,225 × 2	125,125	5,513,056,250	46,46	39,996,389(91%)	17,100,194 × 2(78%)	20,859


*PE, paired-end reads; FPKM, fragments per kilobase of exon per million fragments mapped.*

Pairs of reads with a unique location were then used to estimate genes expression levels. Read counts were normalized as FPKM (Mortazavi et al., 2008) and genes with a minimum expression threshold corresponding to FPKM > 3 were kept. In total, 20,017 and 20,859 genes were considered as expressed in the libraries of Viridiflora and Old Blush, respectively (**Table 1**). The average FPKMs for Viridiflora and Old Blush were 33.18 and 32.57, respectively (**Figure 3B**).

**FIGURE 4:**
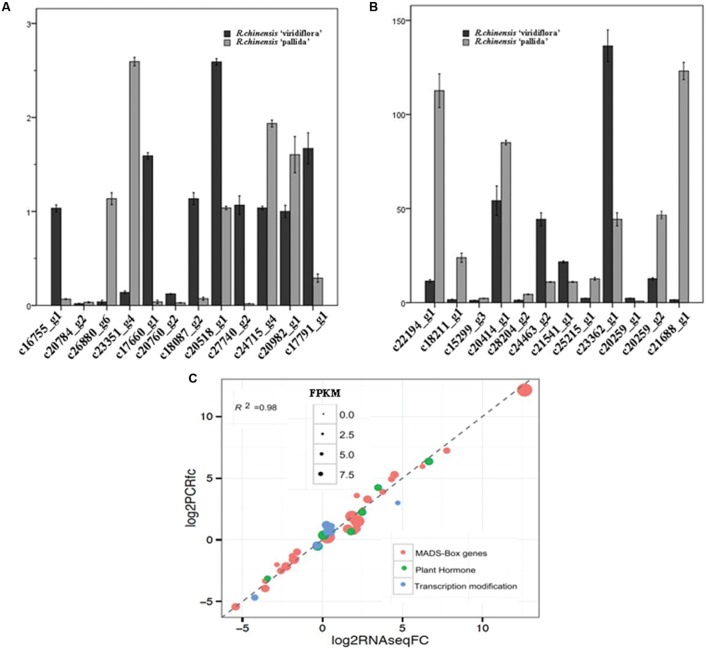
**Expression analyses of selected genes.** **(A,B)** Real time quantitative RT-PCR (qPCR) expression analyses of transcripts selected *in silico*. **(C)** Correlation of qPCR (Y axis) and RNA-Seq (X axis) data for selected genes (involved in flowering and flower organs initiation and development) that exhibited differential expression between in *R. chinensis* cv. Viridiflora with Old Blush.

**FIGURE 5:**
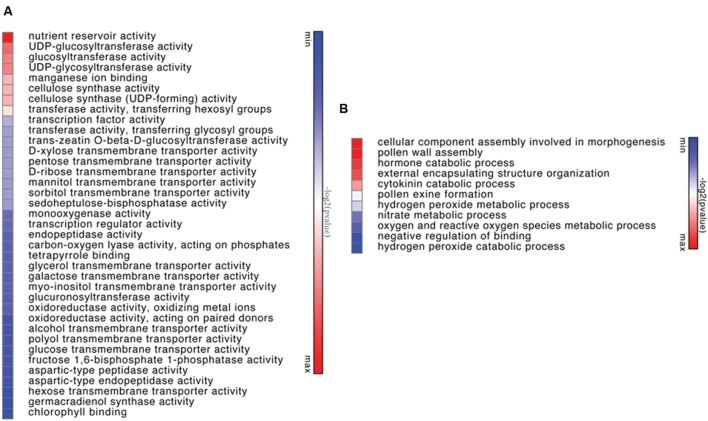
**Heapmat representation of significant functional groups of up-regulated genes in Viridiflora (*P*-value < 0.05) by molecular function **(A)** or by biological function **(B)**.** Colors show the value of log2 (*P*-value), with green representing low *P*-value and red representing high *P*-value.

Genes differentially expressed between of Viridiflora and Old Blush were investigated using the MA-plot-based method with the random sampling model (Wang et al., 2010). 672 unisequences were up-regulated and 666 were down-regulated (**Figure 3C**) in Viridiflora compared to Old Blush, with a fold change of at least 3 and a *P*-value < 0.001 (**Table 1**). Flower development genes that exhibited significant expression difference between Old Blush and Viridiflora were for RT-qPCR validation. Expression of 24 genes among the differentially expressed genes was analyzed using RT-qPCR (**Figures 4A,B**). A good correlation (Pearson correlation coefficient = 0.98) between RT-qPCR data and the RNA-Seq data was observed for the 24 analyzed genes, suggesting that our transcriptomics data are accurate in the different tissues and experimental conditions (**Figure 4C**). GO enrichment analysis showed that transcription factors were enriched in the up-regulated genes fraction, while GO terms associated with ‘pollen wall assembly,’ ‘pollen exine formation,’ and ‘hormone catabolic process’ were enriched in the down-regulated genes fraction (**Figures 5A,B**).

The expressions of the rose homologs of the flower development genes *PI* (c20259_g2), *AG* (c18211_g1), *AGL9* (c26880_g6), *AGL15* (c24715_g4; **Table 2**), Pollen-specific protein *SF3* (Baltz et al., 1992; c15299_g3) and Gibberellin 20 oxidase 1 (c25215_g1; Jacobsen and Olszewski, 1991; Goto and Pharris, 1999) were significantly repressed in Viridiflora compared to Old Blush (**Table 2**). Genes expressions of putative homologs of other flower development transcription factors, such as *AP1* (c16755_g1), *AP2* (c27740_g2), *SOC1* (c24463_g2), *AGL6* (c17791_g1), *AGL8* (c17660_g1; **Table 2**) were in contrast up-regulated in Viridiflora flowers compared to Old Blush flowers. In addition, the bHLH transcription factor UNE10 (Toledo-Ortiz et al., 2003; c18395_g2) and homeotic gene *BEL1* (c20518_g1) were also significantly up-regulated (**Table 2**). BEL1 is known to act as negative regulator of *AG* and has a major role in ovule patterning and in determination of integument identity via its interaction with MADS-box factors (Ray et al., 1994; Mizumoto et al., 2011; Sharma et al., 2014). Taken together these data show that the phyllody phenotype in Viridiflora is mainly associated with modified expression of flowering and flower development related genes.

**Table 2:** Genes known to be involved in flower initiation and development, showing either up- or down-regulated expression levels in *R*. *chinensis* cv. Viridiflora.

Gene ID no.	Description	FPKM fold change	*P*-value
**Up-regulated**			
c24463_g2	MADS-box protein SOC1	7.26	1.89e-11
c17716_g1	Auxin-responsive protein IAA26	10.81	9.79e-33
c18087_g2	MADS-box protein SOC1	11.71	2.97e-21
c16755_g1	Floral homeotic protein APETALA 1	11.91	7.32e-104
c20554_g1	Cytochrome P450	18.81	1.76e-68
c20760_g2	Ethylene-responsive transcription factor ERF105	4.81	3.06e-104
c18395_g2	Transcription factor UNE10	19.88	2.17e-42
c27740_g2	Floral homeotic protein APETALA2	3.43	3.67e-28
c17660_g1	Agamous-like MADS-box protein AGL8	42.98	5.07e-118
c20518_g1	Homeobox protein BEL1	3.68	1.65e-12
c18389_g2	Auxin-binding protein ABP19	83.77	0
c17791_g1	Agamous-like MADS-box protein AGL6	6.03	4.61e-33
**Down-regulated**			
c18211_g1	Floral homeotic protein AGAMOUS	13.63	1.20e-43
c19619_g1	Transcription repressor MYB4	218.60	2.55e-49
c26880_g6	Agamous-like MADS-box protein AGL9	20.10	1.59e-28
c24715_g4	ABC transporter G family member 15	3.01	5.94e-130
c20259_g2	Floral homeotic protein PI/GLO	3.59	0
c15299_g3	Pollen-specific protein SF3	4.36	8.21e-25
c21688_g1	Gibberellin 20 oxidase 1	100.50	3.24e-144
c22194_g2	Floral homeotic protein AGAMOUS	7.10	4.99e-38
c20259_g1	Floral homeotic protein FBP1	4.59	0
c19919_g1	Transcription factor MYB44	76.32	7.49e-20
c28204_g2	Agamous-like MADS-box protein AGL15	3.60	3.86e-15
c20414_g1	Auxin response factor 8	4.0	4.03e-05
c20982_g1	IAA-amino acid hydrolase ILR1	3.43	4.51e-48
c25215_g2	Transcriptional corepressor LEUNIG	17.27	6.95e-13
c18107_g1	Stamen-specific protein FIL1	6421.30	0
c22194_g1	Floral homeotic protein AGAMOUS	7.11	3.86e-54


### Expression Profiles of MADS-Box Transcription Factor Coding Genes

To further investigate the origin of the phyllody phenotype, the expression of MADS-box flower organ identity genes *RcAP1* (c16755_g1), *RcAP2* (c27740_g2), *RcPI* (c20259_g2), *RcAP3* (RC000216; Dubois et al., 2012), *RcAG* (c18211_g1), *RcSEP1* (RC001958; Dubois et al., 2012), and *RcSEP3* (RC000799; Dubois et al., 2012) and flowering *RcSOC1* (c24463_g2) were analyzed using RT-qPCR in leaves and in sepals, petals, stamens, and pistils whorls of developing flowers of Viridiflora and Old Blush.

**FIGURE 6:**
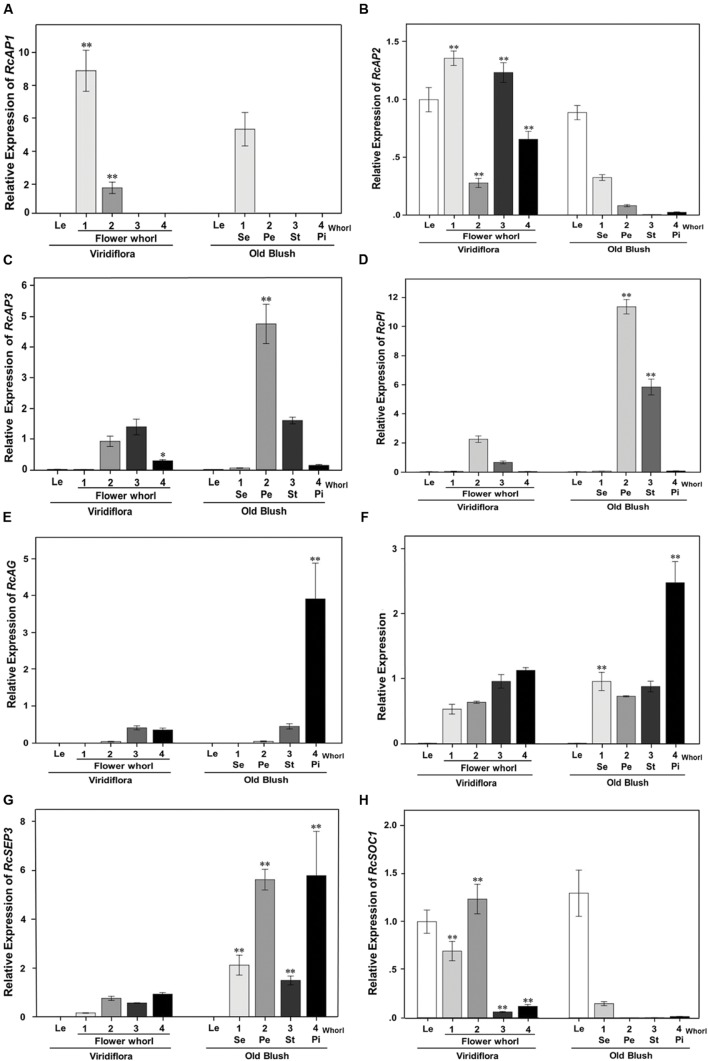
**Quantitative RT-PCR expression analyses of genes involved in flowering and in flower organ identity determination and development.** Expression of *RcAP1*(c16755_g1; **A)**, *RcAP2* (c27740_g2; **B)**, *RcAP3* (RC000216; **C)**, *RcPI* (c20259_g2; **D)**, *RcAG* (c18211_g1; **E)**, *RcSEP1*(RC001958; **F)**, *RcSEP3* (RC000799; **G)**, and *RcSOC1*(c24463_g2; **H)** was analyzed in developing flower buds, at stage 8–10 mm, of Viridiflora and Old Blush. Le, leaves; 1–4, Flower whorls; Se, sepals; Pe, petal; St, stamens; Pi, pistils. Values are mean ± standard deviation (*n* = 3). Asterisks indicate significant differences calculated using Tukey’s test (^∗∗^*P* < 0.01; ^∗^*P* < 0.05).

In wild-type flowers of Old Blush, the A-class genes *RcAP1* mRNA (c16755_g1) accumulated to high levels in whorl 1 (sepals) and showed very low or no expression in whorls 2 (petals), 3 (stamens), and 4 (pistils; **Figure 6A**), thus in agreement with previously published data in *Arabidopsis* (Gustafson-Brown et al., 1994). mRNA of the A-class *RcAP2* was expressed in whorls 1 and 2 of Old Blush (sepal and petal, respectively; **Figure 6B**) and showed relative low expression level in the fourth whorls (pistil; **Figure 6B**), thus similar to previously reported data in *Arabidopsis* (Jofuku et al., 1994). The B-class genes *RcPI* and *RcAP3* were expressed in second and third whorls (petals and stamens respectively) of Old Blush (**Figure 6C**), thus similar pattern as their *Arabidopsis* counterparts, *PI* and *AP3* (Krizek and Meyerowitz, 1996). As expected, the C-class gene *RcAG* (c18211_g1) was expressed in stamens (whorl 3) and in pistils (whorl 4) of Old Blush. The organ identity *RcSEP1* and *RcSEP3* were expressed in all floral whorls (**Figures 6F,G**). In *Arabidopsis*, *SEP1* is expressed in all whorls while SEP3 is expressed in whorls 2, 3, and 4. These data together suggest that the ABCE genetic model of floral organ identity determination is likely conserved in the rose.

In Viridiflora flowers, the expression of *RcAP1* was significantly increased in the sepal and petal whorls (**Figure 6A**). *RcAP1* expression was about 1.7 times induced in whorl 1 and strikingly 20-fold induced in whorls 2 compared to that in Old Blush. The expression of *RcAP2* was up-regulated in all whorls of Viridiflora flowers. Interestingly, the expression of *RcAP2* was significantly up-regulated in stamens and pistils whorls of Viridiflora (**Figure 6B**) and conversely the expression of the C-class gene *RcAG* was significantly down-regulated in whorl 4 compared to that in Old Blush (**Figure 6E**). It should be noted that *RcAG* was about eightfold over-expressed in the pistils compared to stamens (**Figure 6E**). These data are also in agreement with the antagonist effect between A and C functions, known as crucial in floral patterning (Gustafson-Brown et al., 1994). Furthermore, *RcSEP3* mRNA was about sixfold under-expressed in all flower whorls of Viridiflora compared to Old Blush (**Figure 6G**). However, the expression of *RcSEP1* was not strikingly different between the two roses, except for a relative low expression level in whorl 4 (pistils) of Viridiflora compared to Old Blush (**Figure 6F**). The phyllody phenotype in Viridiflora was also associated with significant down-regulation of the B class genes *RcP1* and *RcAP3* in the petals and stamens whorls (2 and 3, respectively) compared to Old Blush (5- and 6-fold, respectively; **Figures 6C,D**).

Interestingly, in Viridiflora, the expression of the flowering time integrator MADS-box *RcSOC1* gene was induced in petals, stamens and pistils whorls, with the strongest expression in sepals and in petals whorls (**Figure 6H**). In Old Blush, the expression of *RcSOC1* was confined to leaves and although at lower levels to sepals as well, thus a similar pattern as in *Arabidopsis* (Samach et al., 2000).

To summarize, these data show that in whorl 2, the loss of petal identity is consistent with the down-regulation of the B (*RcPI* and *RcAP3*) and E-class (*RcSEP3*) organ identity genes. The loss of petal organ identity combined with the strong up-regulation of *RcSOC1* is likely associated with the conversion of petals into leaf-like structures in whorl 2. In whorls 3 and 4, the ectopic expression of the A-class *RcAP2* and the down-regulation of the E-class *RcSEP3* in combination with the down regulation of B-class genes *RcPI* and *RcAP3* in whorl 3 and of the C-class *RcAG* in whorl 4 is consistent with the observed loss of stamens and pistils identity, respectively, and their conversion into leaf-like structures.

## Discussion

Phyllody is a flower abnormality in which leaf-like structures replace flower organs. Two types of phyllody were described in the genus *Rosa*. The first type of phyllody (*R*. *chinensis* cv. Viridiflora) is a stable mutation known as the ‘green rose’ characterized by the transformation of all flower organs to leaf-like structures (Krussman, 1981). For the second type, described in *R. x hybrida* cv. Motrea, phyllody is restricted to reproductive organs (third and fourth whorls; Mor and Zieslin, 1992). It was suggested that Viridiflora phenotype might be caused by phytoplasma infection (McCoy et al., 1989; Szyndel, 2003; Zhang and Zhu, 2006). Here we used grafting, transmission electron microscopy and PCR experiments, to demonstrate that phyllody phenotype in Viridiflora is not associated with phytoplasma infection (**Figure 2**).

**FIGURE 7:**
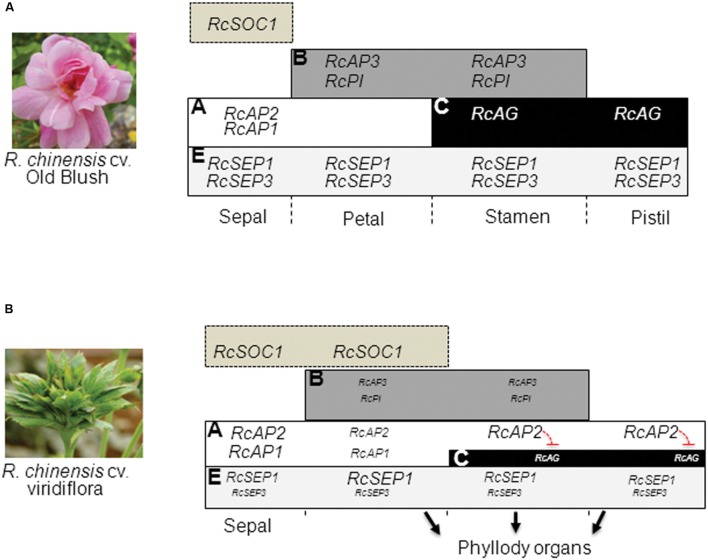
**Model for Viridiflora phenotype formation.** **(A)** wild-type ABCE model and expression of flower organ identity genes. **(B)** In Viridiflora the conversion of petals into leaf-like structures in whorl two is associated with the down-regulation of petal organ identity genes *RcAP3*, *RcPI*, and *RcSEP3* combined with up-regulation of *RcSOC1*. In the third and fourth whorls the conversion of stamens and pistils to leaf-like structures is associated with a shift of A-class gene *RcAP2* expression domain together with down-regulation of E class gene *RcSEP3* and down-regulation of B (*RcAP3* and *RcPI*), and C (*RcAG*). The down-regulation of gene expression is indicated with text of smaller size.

Gene expression analysis in flowers early during development, showed that the phyllody phenotype in Viridiflora is associated with ectopic expression of the flowering time gene *RcSOC1* and with misexpression of the ABCE flower organ identity genes leading to loss of petal, stamen and pistil organ identity (**Figure 7**). These data indicate that in Viridiflora a shift of the expression of the A-class gene *RcAP2* toward the third and fourth whorls together with significantly reduced expression of B, C, and E gene classes are associated with the observed phyllody phenotype. These data also highlights the antagonist effect of the A and C class gene.

The A-class gene *AP1* is known to be involved in sepal and petal organ identity determination. In *R. chinensis* cv. Old Blush, *RcAP1* showed high expression levels in developing sepals and low expression levels in developing petals (**Figure 6A**). Similar data were observed in *R. hybrida* (Mibus et al., 2011) and in other flowering plant species (Huijser et al., 1992; Mandel et al., 1992). Interestingly, we observed a strong down-regulation of the B-class genes *RcPI* and *RcAP3* in petals and stamens whorls and of the E class gene *RcSEP3* in all whorls. In *Arabidopsis* genetic and gene expression experiments showed that SEP3 is required for the up-regulation of B and C floral homeotic genes (Castillejo et al., 2005; Kaufmann et al., 2009). It is possible that the downregulation of *RcAP3* and *RcPI* is a result of reduced expression of *RcSEP3*. In the flowering plants, mutation of the B-class genes leads to conversion of petals into sepals. In Viridiflora the down-regulation of *RcPI* and *RcAP3* cannot explain the leaf-like phenotype of stamens in Viridiflora. It is likely that the up-regulation of SOC1 in the context of the B (*RcPI* and *RcAP3*) and E (*RcSEP3*) genes down-regulation are at least in part at the origin of the loss of petal identity and phyllody phenotype. In whorl 3, the up-regulation of *RcAP2* and the down regulation of *RcPI*, *RcAP3* and *RcSEP3* are likely at the origin of loss of stamen identity and phylloid organs formation.

*AGAMOUS* (AG), the C-class floral homeotic gene is involved in the specification of stamens and pistils identity and the termination of the floral meristem in the center of the flower (Kapoor et al., 2002; ÓMaoiléidigh et al., 2013). In Viridiflora, our data show that mRNA of *RcAG* was down-regulated in stamens and pistils, compared to Old Blush (**Figure 6D**). In *Arabidopsis* the down-regulation of *AG* leads to conversion of stamens into petals (Yanofsky et al., 1990). Similarly, the silencing of pMADS3, a *petunia* C-class gene, resulted in homeotic conversion of stamens into petaloid structures (Kapoor et al., 2002). Interestingly, in the ranunculid *Thalictrum thalictroides*, down-regulation of the *AG* homolog *ThtAG1* by the mean of virus-induced gene silencing resulted in homeotic conversion of stamens and pistils into sepaloid organs and loss of flower determinacy (Galimba et al., 2012). We observed that the putative homolog of *BEL1* is up-regulated in Viridiflora flowers. In *Arabidopsis BEL1* was shown to act as negative regulator of *AG* (Ray et al., 1994; Sharma et al., 2014). Therefore, we cannot rule out that the down-regulation of *RcAG* could be a consequence of increased *RcBEL1* levels. In addition, *RcAP2* showed ectopic expression in stamens and pistils of Viridiflora (**Figure 6B**). This result was not surprising, as it is known that *AP2* is a negative regulator of *AG* expression in the first two whorls of the flower (Drews et al., 1991). Therefore, it is likely that in Viridiflora the ectopic expression of *RcAP2* in stamens and pistils whorls lead to the observed down-regulation of *RcAG* expression in whorl 4. It has been reported in *Arabidopsis* that *AP2*, besides its antagonist role with the C-class gene *AG*, is also involved in repressing B-class genes *AP3* and *PI* by regulating their outer boundary expression (Krogan et al., 2012; Wuest et al., 2012). It is likely that similar role is conserved in the rose and that in Viridiflora the up-regulation of *RcAP2* in whorl 3 (**Figure 6B**) is responsible for the observed down-regulation of the B-class genes *RcAP3* and *RcPI* (**Figures 6C,D**) and thus loss of petal and stamens organ identity.

We show that the phyllody phenotype in Viridiflora is associated with ectopic expression of the putative homolog of the flowering time integrator gene *SOC1.* In *Arabidopsis SOC1* plays a central role to integrate the photoperiodic, the autonomous, the vernalization, and the gibberellin pathways during flowering (Borner et al., 2000; Samach et al., 2000; Moon et al., 2003). SOC1-like genes are preferentially expressed in vegetative parts of both angiosperms and gymnosperms (Tandre et al., 1995; Winter et al., 1999; Watson and Brill, 2004), but examples of SOC1-like genes expressed in reproductive organs have also been reported (Heuer et al., 2001). *AtSOC1* is mainly expressed in meristems and developing leaves (Samach et al., 2000). Over-expression of *AtSOC1*-like genes causes early flowering in several plant species (Tadege et al., 2003; Ferrario et al., 2004; Smykal et al., 2007). Interestingly, it has also been shown that over-expression of *GhSOC1* in gerbera leads to a partial loss of floral organ identity, but did not affect flowering time (Ruokolainen et al., 2010, 2011). In *petunia*, ectopic expression of *UNSHAVEN* (*petunia SOC1* homolog) leads to conversion of petals to organs with leaf-like features (Ferrario et al., 2004). Here, we also show that in the rose similar conversion of petals to leaf-like structures is also associated with ectopic expression of *RcSOC1*. In *Arabidopsis SOC1* has been shown to regulate the flower meristem identity gene *LEAFY* (*LFY*; Lee et al., 2008). LFY links floral induction and flower organs identity determination and development via induction of the ABC genes. Whether in the rose *RcSOC1* acts in similar pathway this remains to be addressed.

## Conclusion

Taken together, our data with those in the literature suggest that the phyllody phenotype in the rose Viridiflora is associated with an up-regulation and ectopic expression of *RcSOC1* and of the A-class flower organ identity genes along with the down-regulation of the B, C, and E floral organ identity genes. Therefore, these data here represent a base for future research to deeply understand the molecular mechanisms associated with ABCE organ identity genes, and the origin of type 1 phyllody phenotype in roses.

## Author Contributions

HY, MB, and KT designed the entire research. HY, HZ, QW, HJ, and XQ performed experiments and helped to analyze data. LG and OR analyzed transcriptome data. SB, JJ, and JW revised the manuscript. HY and MB wrote and edited the manuscript. All authors have read and approved the final manuscript.

## Conflict of Interest Statement

The authors declare that the research was conducted in the absence of any commercial or financial relationships that could be construed as a potential conflict of interest.

## Footnotes

1 http://bioinfor.org/pub/Viridiflora.

2 http://bioinfo.ut.ee/primer3/.

## Supplementary Material

**FIGURE S1 | Electron-microscopic analysis of sap extracts from young buds at stage 8–10 mm of *R. chinensis* cv. Viridiflora **(A)** and Old Blush used as negative control **(B)**.** The samples were negatively stained and analyzed at a magnification of ×100 000. The bar represents 100 nm. No trace of mycoplasma is observed in Viridiflora or Old Blush samples.

**FIGURE S2 | No trace of MLO DNA is found in Viridiflora and Old Blush: DNA was prepared from buds at stage 8–10 mm of *R. chinensis* Viridiflora and Old Blush and then used to PCR amplify MLO DNA.** M, molecular marker; C, positive control using MLO plasmid; 1,Viridiflora DNA; 2,Old Blush DNA.

**TABLE S1 | Primers used for real-time quantitative PCR.**

**DATA SHEET S1 | The sequences of genes used for qRT-PCR validation.**

## References

[B1] Aldaghi M. Massart S. Roussel S. Steyer S. Lateur M. Jijakli M. H. (2007). Comparison of different techniques for inoculation of “Candidatus Phytoplasma mali” on apple and periwinkle in biological indexing procedure. *Commun. Agric. Appl. Biol. Sci.* 72 779–784. 18396810

[B2] Baltz R. Domon C. Pillay D. T. N. Steinmetz A. (1992). Characterization of a pollen-specific cDNA from sunflower encoding a zinc finger protein. *Plant J.* 2 713–721. 10.1046/j.1365-313X.1992.t01-13-00999.x 1302629

[B3] Bendahmane M. Dubois A. Raymond O. Le Bris M. (2013). Genetics and genomics of flower initiation and development in roses. *J. Exp. Bot.* 64 847–857. 10.1093/jxb/ers387 23364936 PMC3594942

[B4] Borner R. Kampmann G. Chandler J. Gleissner R. Wisman E. Apel K. (2000). A MADS domain gene involved in the transition to flowering in *Arabidopsis*. *Plant J.* 24 591–599. 10.1046/j.1365-313x.2000.00906.x 11123798

[B5] Castillejo C. Romera-Branchat M. Pelaz S. (2005). A new role of the *Arabidopsis* SEPALLATA3 gene revealed by its constitutive expression. *Plant J.* 43 586–596. 10.1111/j.1365-313X.2005.02476.x 16098111

[B6] Chmelmitsky I. Azizbekova N. Khayat E. Zieslin N. (2002). Morphological development of normal and phyllody expressing *Rosa hybrida* cv. Motrea flowers. *J. Plant Growth Regul.* 37 215–221. 10.1023/A:1020819123385

[B7] Coen E. S. Meyerowitz E. M. (1991). The war of the whorls: genetic interactions controlling flower development. *Nature* 353 31–37. 10.1038/353031a0 1715520

[B8] Drews G. N. Bowman J. L. Meyerowitz E. M. (1991). Negative regulation of the *Arabidopsis* homeotic gene *AGAMOUS* by the *APETALA2* product. *Cell* 65 991–1002. 10.1016/0092-8674(91)90551-9 1675158

[B9] Dubois A. Carrere S. Raymond O. Pouvreau B. Cottret L. Roccia A. (2012). Transcriptome database resource and gene expression atlas for the rose. *BMC Genomics* 13:638 10.1186/1471-2164-13-638 PMC3518227 23164410

[B10] Dubois A. Raymond O. Maene M. Baudino S. Langlade N. B. Boltz V. (2010). Tinkering with the C-function: a molecular frame for the selection of double flowers in cultivated roses. *PLoS ONE* 5:e9288 10.1371/journal.pone.0009288 PMC2823793 20174587

[B11] Dubois A. Remay A. Raymond O. Balzergue S. Chauvet A. Maene M. (2011). Genomic approach to study floral development genes in *Rosa* sp. *PLoS ONE* 6:e28455 10.1371/journal.pone.0028455 PMC3237435 22194838

[B12] Ferrario S. Busscher J. Franken J. Gerats T. Vandenbussche M. Angenent G. C. (2004). Ectopic expression of the petunia MADS box gene UNSHAVEN accelerates flowering and confers leaf-like characteristics to floral organs in a dominant-negative manner. *Plant Cell* 16 1490–1505. 10.1105/tpc.019679 15155884 PMC490041

[B13] Galimba K. D. Tolkin T. R. Sullivan A. M. Melzer R. Theißen G. Di Stilio V. S. (2012). Loss of deeply conserved C-class floral homeotic gene function and C-and E-class protein interaction in adouble-flowered ranunculid mutant. *Proc. Natl. Acad. Sci. U.S.A.* 109 2267–2275. 10.1073/pnas.1203686109 PMC3427126 22853954

[B14] Goldschmidt E. E. (2014). Plant grafting: new mechanisms, evolutionary implications. *Front. Plant Sci.* 5:727 10.3389/fpls.2014.00727 PMC4269114 25566298

[B15] Golino D. A. Oldfield G. N. Gumpf D. (1989). Experimental hosts of the beet leaf hopper-transmitted virescence agent. *Plant Dis.* 73 850–854. 10.1094/PD-73-0850

[B16] Goto N. Pharris R. P. (1999). Role of gibberellins in the development of floral organs of the gibberellin-deficient mutant ga1-1 of *Arabidopsis thaliana*. *J. Bot.* 77 944–954. 10.1139/cjb-77-7-944

[B17] Grabherr M. G. Haas B. J. Yassour M. Levin J. Z. Thompson D. A. Amit I. (2011). Full-length transcriptome assembly from RNA-Seq data without a reference genome. *Nat. Biotechnol.* 29 644–652. 10.1038/nbt.1883 21572440 PMC3571712

[B18] Gustafson-Brown C. Savidge B. Yanofsky M. F. (1994). Regulation of the *Arabidopsis* floral homeotic gene APETALA1. *Cell* 76 131–143. 10.1016/0092-8674(94)90178-3 7506995

[B19] Heijmans K. Morel P. Vandenbussche M. (2012). MADS-box genes and floral development: the dark side. *J. Exp. Bot.* 63 5397–5404. 10.1093/jxb/ers233 22915743

[B20] Heuer S. Hansen S. Bantin J. Brettschneider R. Kranz E. Lörz H. (2001). The maize MADS box gene ZmMADS3 affects node number and spikelet development and is co-expressed with ZmMADS1 during flower development, in egg cells, and early embryogenesis. *Plant Physiol.* 127 33–45. 10.1104/pp.127.1.33 11553732 PMC117960

[B21] Hibino Y. Kitahara K. Hirai S. Matsumoto S. (2006). Structural and functional analysis of rose class B MADS-box genes ‘MASAKO BP, euB3 and B3’: paleo-type AP3 homologue ‘MASAKO B3’ association with petal development. *Plant Sci.* 170 778–785. 10.1016/j.plantsci.2005.11.010

[B22] Hogenhout S. A. Oshima K. Ammar E.-D. Kakizawa S. Kingdom H. Namba S. (2008). Phytoplasmas: bacteria that manipulate plants and insects. *Mol. Plant Pathol.* 9 403–423. 10.1111/j.1364-3703.2008.00472.x 18705857 PMC6640453

[B23] Huijser P. Klein J. Lonnig W. E. Meijer H. Saedler H. Sommer H. (1992). Bracteomania, an inflorescence anomaly, is caused by the loss of function of the MADS-box gene squamosa in *Antirrhinum majus*. *EMBO J.* 11 1239–1249. 1563342 10.1002/j.1460-2075.1992.tb05168.x PMC556572

[B24] Jacobsen S. E. Olszewski N. E. (1991). Characterization of the arrest in anther development associated with gibberellin deficiency of the gib-1 mutant of tomato. *Plant Physiol.* 97 409–414. 10.1104/pp.97.1.409 16668400 PMC1081013

[B25] Jofuku K. D. den Boer B. G. W. Montagu M. V. Okamuro J. K. (1994). Control of *Arabidopsis* flower and seed development by the homeotic gene APETALA2. *Plant Cell* 6 1211–1225. 10.1105/tpc.6.9.1211 7919989 PMC160514

[B26] Kapoor M. Tsuda S. Tanaka Y. Mayama T. Okuyama Y. Tsuchimoto S. (2002). Role of petunia pMADS3 in determination of floralor-gan andmeristem identity, as revealed by its loss of function. *Plant J.* 32 115–127. 10.1046/j.1365-313X.2002.01402.x 12366805

[B27] Kaufmann K. Muiño J. M. Jauregui R. Airoldi C. A. Smaczniak C. Krajewski P. (2009). Target genes of the MADS transcription factor SEPALLATA3: integration of developmental and hormonal pathways in the *Arabidopsis* flower. *PLoS Biol.* 7:e1000090 10.1371/journal.pbio.1000090 PMC2671559 19385720

[B28] Kitahara K. Hibino Y. Aida R. Matsumoto S. (2004). Ectopic expression of the rose AGAMOUS-like MADS-box genes ‘MASAKOC1 and D1’ causes similar homeotic transformation of sepal and petal in *Arabidopsis* and sepal in Torenia. *Plant Sci.* 166 1245–1252. 10.1016/j.plantsci.2003.12.040

[B29] Kitahara K. Matsumoto S. (2000). Rose MADS-box genes ‘MASAKOC1 and D1’ homologous to class C floral identity genes. *Plant Sci.* 151 121–134. 10.1016/S0168-9452(99)00206-X 10808068

[B30] Krizek B. A. Meyerowitz E. M. (1996). The *Arabidopsis* homeotic genes APETALA3 and PISTILLATA are sufficient to provide the B class organ identity function. *Development* 122 11–22. 8565821 10.1242/dev.122.1.11

[B31] Krogan N. T. Hogan K. Long J. A. (2012). APETALA2 negatively regulates multiple floral organ identity genes in *Arabidopsis* by recruiting the co-repressor TOPLESS and the histone deacetylase HDA19. *Development* 139 4180–4190. 10.1242/dev.085407 23034631 PMC3478687

[B32] Krussman G. (1981). *The Complete Book of Roses.* Portland: Timber Press.

[B33] Ku T. C. Robertson K. R. (2003). “*Rosa* (*Rosa*ceae),” in *Flora of China*, eds Wu Z. Y. Raven P. H. (Beijing: Science Press), 339–380.

[B34] Langmead B. Salzberg S. L. (2012). Fast gapped-read alignment with Bowtie. *Nat. Methods* 9 357–359. 10.1038/nmeth.1923 22388286 PMC3322381

[B35] Lee I. M. Hammond R. W. Davis R. E. Gundersen D. E. (1993). Universal amplification and analysis of pathogen 16S rDNA for classification and identification of mycoplasmalike organisms. *Phytopathology* 83 834–842. 10.1094/Phyto-83-834

[B36] Lee J. Oh M. Park H. Lee I. (2008). SOC1 translocated to the nucleus by interaction with AGL24 directly regulates LEAFY. *Plant J.* 55 832–843. 10.1111/j.1365-313X.2008.03552.x 18466303

[B37] Liu Z. M. (1964). Roses of china and roses of Europe. *Acta Hortic. Sin.* 3 387–394.

[B38] Livak K. J. Schmittgen T. D. (2001). Analysis of relative gene expression data using real-time quantitative PCR and the 2^-ΔΔ*C*_T_^ method. *Methods* 25 402–408. 10.1006/meth.2001.1262 11846609

[B39] Ma N. Chen W. Fan T. Tian Y. Zhang S. Zeng D. (2015). Low temperature-induced DNA hypermethylation attenuates expression of RhAG, an AGAMOUS homolog, and increases petal number in rose (*Rosa hybrida*). *BMC Plant Biol.* 15:237 10.1186/s12870-015-0623-1 PMC4595006 26438149

[B40] Maejima K. Iwai R. Himeno M. Komatsu K. Kitazawa Y. Fujita N. (2014). Recognition of floral homeotic MADS domain transcription factors by a phytoplasmal effector, phyllogen, induces phyllody. *Plant J.* 78 541–554. 10.1111/tpj.12495 24597566 PMC4282529

[B41] Maejima K. Kitazawa Y. Tomomitsu T. Yusa A. Neriya Y. Himeno M. (2015). Degradation of class E MADS-domain transcription factors in *Arabidopsis* by a phytoplasmal effector, phyllogen. *Plant Signal. Behav.* 10:e1042635 10.1080/15592324.2015.1042635 PMC4623417 26179462

[B42] Mandel M. A. Bowman J. L. Kempin S. A. Ma H. Meyerowitz E. M. Yanofsky M. F. (1992). Manipulation of flower structure in transgenic tobacco. *Cell* 71 133–143. 10.1016/0092-8674(92)90272-E 1356631

[B43] Martin M. Piola F. Chessel D. Jay M. Heizmann P. (2001). The domestication process of the Modern Rose: genetic structure and allelic composition of the rose complex. *Theor. Appl. Genet.* 102 398–404. 10.1007/s001220051660

[B44] McCoy R. E. Caudwell A. Chang C. J. Chen T. A. Chen T. Y. Chiykowski M. T. (1989). “Plant diseases associated with mycoplasmas,” in *The Mycoplasmas*, eds Whitcomb R. F. Tully J. G. (New York, NY: Academic Press), 546–640.

[B45] Meyer V. G. (1966). Flower abnormality. *Bot. Rev.* 32 165–195. 10.1007/BF02858659

[B46] Mibus H. Heckl D. Serek M. (2011). Cloning and characterization of three APETALA1/FRUITFULL-like genes in different flower types of *Rosa* × *hybrida* L. *J. Plant Growth Regul.* 30 272–285. 10.1007/s00344-010-9190-8

[B47] Mizumoto K. Hatano H. Hirabayashi C. Murai K. Takumi S. (2011). Characterization of wheat bell1-type homeobox genes in floral organs of alloplasmic lines with *Aegilops crassa* cytoplasm. *BMC Plant Biol.* 11:2 10.1186/1471-2229-11-2 PMC3022553 21205321

[B48] Moon J. Suh S. S. Lee H. Choi K. R. Hong C. B. Paek N. C. (2003). The SOC1 MADS-box gene integrates vernalization and gibberellin signals for flowering in *Arabidopsis*. *Plant J.* 35 613–623. 10.1046/j.1365-313X.2003.01833.x 12940954

[B49] Mor Y. Zieslin N. (1992). Phyllody malformation in flowers of *Rosa* × *hybrida* cv. Motrea: effects of rootstocks, flower position, growth regulators and season. *J. Exp. Bot.* 43 89–93. 10.1093/jxb/43.1.89

[B50] Mortazavi A. Williams B. A. McCue K. Schaeffer L. Wold B. (2008). Mapping and quantifying mammalian transcriptomes by RNA-Seq. *Nat. Methods* 5 621–628. 10.1038/nmeth.1226 18516045 PMC13303166

[B51] Ohkawa K. (1980). Cutting-grafts as a means to propagate greenhouse roses. *Sci. Hortic.* 13 191–199. 10.1016/0304-4238(80)90084-9

[B52] ÓMaoiléidigh D. S. Wuest S. E. Rae L. Raganelli A. Ryan P. T. Kwasniewska K. (2013). Control of reproductive floral organ identity specification in *Arabidopsis* by the C function regulator AGAMOUS. *Plant Cell* 25 2482–2503. 10.1105/tpc.113.113209 23821642 PMC3753378

[B53] Pelaz S. Ditta G. S. Baumann E. Wisman E. Yanofsky M. F. (2000). B and C floral organ identity functions require SEPALLATA MADS-box genes. *Nature* 405 200–203. 10.1038/35012103 10821278

[B54] Ray A. Robinson-Beers K. Ray S. Baker S. C. Lang J. D. Preuss D. (1994). *Arabidopsis* floral homeotic gene BELL (BEL1) controls ovule development through negative regulation of AGAMOUS gene (AG). *Proc. Natl. Acad. Sci. U.S.A.* 91 5761–5765. 10.1073/pnas.91.13.5761 7912435 PMC44076

[B55] Ruokolainen S. Ng Y. P. Albert V. A. Elomaa P. Teeri T. H. (2010). Large scale interaction analysis predicts that the *Gerbera hybrida* floral E function is provided both by general and specialized proteins. *BMC Plant Biol.* 10:129 10.1186/1471-2229-10-129 PMC3017775 20579338

[B56] Ruokolainen S. Ng Y. P. Albert V. A. Elomaa P. Teeri T. H. (2011). Over-expression of the *Gerbera hybrida* At-SOC1-like1 gene Gh-SOC1 leads to floral organ identity deterioration. *Ann. Bot.* 107 1491–1499. 10.1093/aob/mcr112 21572092 PMC3108810

[B57] Samach A. Onouchi H. Gold S. E. Ditta G. S. Schwarz-Sommer Z. Yanofsky M. F. (2000). Distinct roles of CONSTANS target genes in reproductive development of *Arabidopsis*. *Science* 288 1613–1616. 10.1126/science.288.5471.1613 10834834

[B58] Sharma P. Lin T. Grandellis C. Yu M. Hannapel D. J. (2014). The BEL1-like family of transcription factors in potato. *J. Exp. Bot.* 65 709–723. 10.1093/jxb/ert432 24474812 PMC3904721

[B59] Smaczniak C. Immink R. G. H. Angenent G. C. Kaufmann K. (2012). Developmental and evolutionary diversity of plant MADS-domain factors: insights from recent studies. *Development* 139 3081–3098. 10.1242/dev.074674 22872082

[B60] Smykal P. Gennen J. De Bodt S. Ranganath V. Melzer S. (2007). Flowering of strict photoperiodic *Nicotiana* varieties in non-inductive conditions by transgenic approaches. *Plant Mol. Biol.* 65 233–242. 10.1007/s11103-007-9211-6 17660946

[B61] Szyndel M. S. (2003). “Viruses,” in *the Encyclopedia of Rose Science*, eds Roberts A. V. Debener T. Gudin S. (Oxford: Elsevier Academic Press), 180–190.

[B62] Tadege M. Sheldon C. C. Helliwell C. A. Upadhyaya N. M. Dennis E. S. Peacock W. J. (2003). Reciprocal control of flowering time by OsSOC1 in transgenic *Arabidopsis* and by FLC in transgenic rice. *Plant Biotechnol. J.* 1 361–369. 10.1046/j.1467-7652.2003.00034.x 17166135

[B63] Tandre K. Albert V. A. Sundas A. Engstrom P. (1995). Conifer homologues to genes that control floral development in angiosperms. *Plant Mol. Biol.* 27 69–78. 10.1007/BF00019179 7865797

[B64] Toledo-Ortiz G. Huq E. Quail P. H. (2003). The *Arabidopsis* basic/helix-loop-helix transcription factor family. *Plant Cell* 15 1749–1770. 10.1105/tpc.013839 12897250 PMC167167

[B65] Wang L. Feng Z. Wang X. Zhang X. (2010). DEGseq: an R package for identifying differentially expressed genes from RNA-seq data. *Bioinformatics* 26 136–138. 10.1093/bioinformatics/btp612 19855105

[B66] Watson J. M. Brill E. M. (2004). Eucalyptus grandis has at least two functional SOC1-like floral activator genes. *Funct. Plant Biol.* 31 225–234. 10.1071/FP03181 32688894

[B67] Weigel D. Meyerowitz E. M. (1994). The ABC of floral homeotic genes. *Cell* 78 203–209. 10.1016/0092-8674(94)90291-7 7913881

[B68] Wellmer F. Bowman J. L. Davies B. Ferrándiz C. Fletcher J. C. Franks R. G. (2014). Flower development: open questions and future directions. *Methods Mol. Biol.* 1110 103–124. 10.1007/978-1-4614-9408-9_5 24395254

[B69] Winter K. U. Becker A. Munster T. Kim J. T. Saedler H. Theissen G. (1999). MADS-box genes reveal that gnetophytes are more closely related to conifers than to flowering plants. *Proc. Natl. Acad. Sci. U.S.A.* 96 7342–7347. 10.1073/pnas.96.13.7342 10377416 PMC22087

[B70] Wuest S. E. ÓMaoiléidigh D. S. Rae L. Kwasniewska K. Raganelli A. Hanczaryk K. (2012). Molecular basis for the specification of floral organs by APETALA3 and PISTILLATA. *Proc. Natl. Acad. Sci. U.S.A.* 109 13452–13457. 10.1073/pnas.1207075109 22847437 PMC3421202

[B71] Wylie A. P. (1954). The history of garden roses. *J. R. Hortic. Soc.* 79 555–571.

[B72] Yan H. J. Zhang H. Chen M. Jian H. J. Baudino S. Caissard J. C. (2014). De novo transcriptome analysis and identification of scent-related genes from *Rosa* chinensis ‘Pallida.’ *Gene* 540 96–105. 10.1016/j.gene.2014.02.008 24530310

[B73] Yan H. J. Zhang H. Wang Q. G. Jian H. Y. Xiu X. Q. Wang J. H. (2011). Isolation and identification of a putative scent-related gene RhMYB1 from rose. *Mol. Biol. Rep.* 38 4475–4482. 10.1007/s11033-010-0577-1 21132535

[B74] Yanofsky M. F. Ma H. Bowman J. L. Drews G. N. Feldmann K. A. Meyerowitz E. M. (1990). The protein encoded by the *Arabidopsis* homeotic gene agamous resembles transcription factors. *Nature* 346 35–39. 10.1038/346035a0 1973265

[B75] Yin Y. Y. Zheng K. Y. Dong J. H. Fang Q. Wu S. P. Wang L. S. (2014). Identification of a new tospovirus causing necrotic ringspot on tomato in China. *Virol. J.* 11:213 10.1186/s12985-014-0213-0 PMC4263035 25465801

[B76] Zhang Z. S. Zhu X. Z. (2006). *China Rose.* Beijing: China Forestry Publishing House.

