# Supplementary figures and images for: The Rosa chinensis cv. Viridiflora Phyllody Phenotype Is Associated with Misexpression of Flower Organ Identity Genes
Source: Front Plant Sci. 2016 Jul 12;7:996. doi: 10.3389/fpls.2016.00996 (PMC4941542; doi:10.3389/fpls.2016.00996)

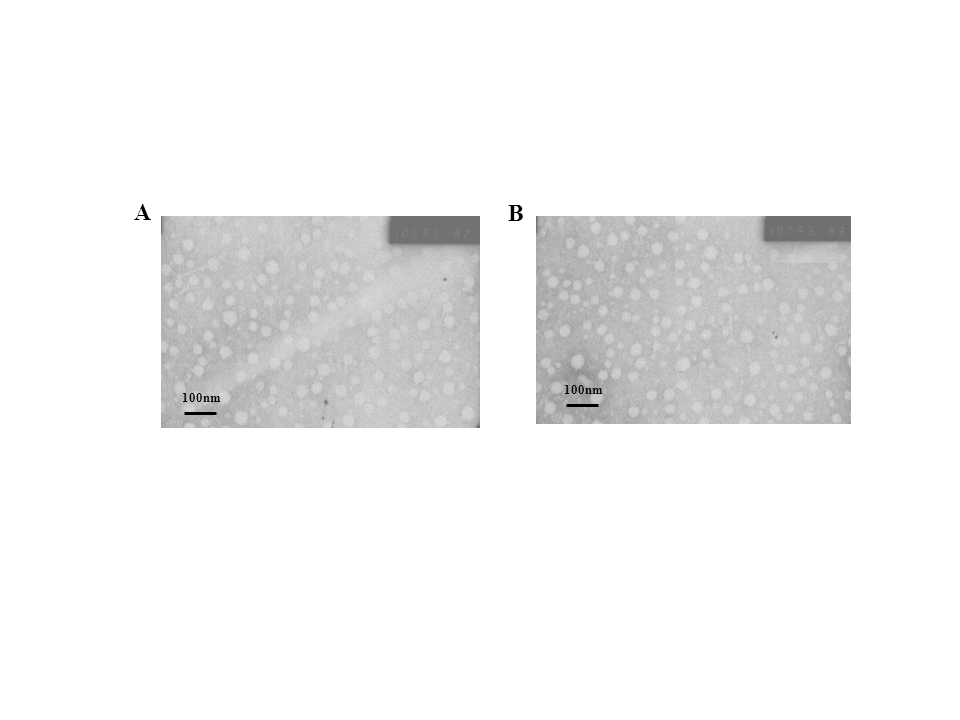

Supplement: Supplementary file 1 [file Image_1.TIF]

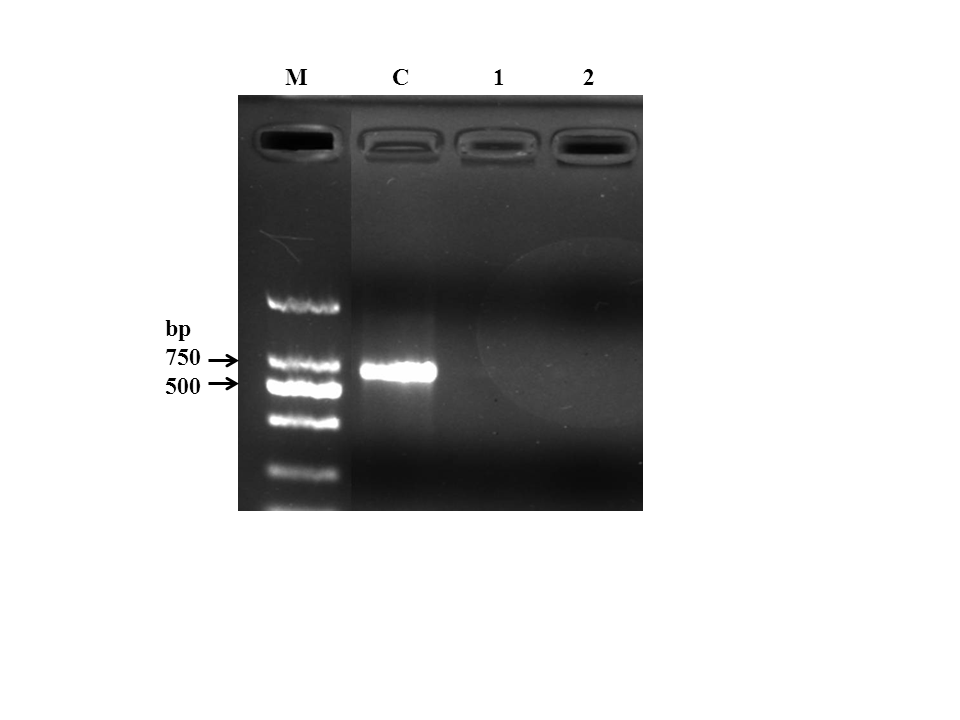

Supplement: Supplementary file 2 [file Image_2.TIF]
