# Supplementary material for: The Rosa chinensis cv. Viridiflora Phyllody Phenotype Is Associated with Misexpression of Flower Organ Identity Genes
Source: Front Plant Sci. 2016 Jul 12;7:996. doi: 10.3389/fpls.2016.00996 (PMC4941542; doi:10.3389/fpls.2016.00996)
Supplement: Supplementary file 3 [file Table_1.DOCX]

| No. | Forward primer | Reverse primer | Production size |
| --- | --- | --- | --- |
| c18211_g1 | AGTCACATTCTGCAAGCGTC | GCATATTCATAGAGGCGCCC | 120 |
| c19619_g1 | TGGGTTACAGGGTCGATTCC | GGAAGCAGGTGGTCTCTGAT | 122 |
| c26880_g6 | GACCTCCGAGCATAGATCTCA | TGTGAAGATGAGTCATACCCCA | 115 |
| c15505_g1 | ACCAACACGACCCTCATCAT | GCTCTTGGGTCCTCACTTCT | 123 |
| c36877_g1 | TAGCAGATCTGGAGGGGTTG | CGGAGTGCCAAAGTTCTTTAAA | 117 |
| c24676_g3 | TGAGATCCCAGCCTGTACTT | TTGAGGAAGGAAGGGAAGGC | 120 |
| c22378_g1 | TCAGAGACAGGTGAAGGAGC | CGGAGATGTATCACAACGGC | 115 |
| c24715_g4 | GATCAGTACAGACGCTCGGA | TGAGAGTTGCTTCCACCACT | 126 |
| c23899_g1 | ATGCTAGTGGGAACTGAGCC | GGTTATGCTGCCAGGGGTTA | 117 |
| c18487_g2 | CGCCAAGACCAACCATGATG | GGCATCAGAAAACCAGCCAT | 115 |
| c19627_g2 | GCACGAATGAAGGGTATGCA | CACCCAACACGTTCCAACAT | 130 |
| c27010_g3 | TCCAGATCCGCTAGCATCAG | CAGCCTCAGAGCAAGCATTT | 116 |
| c20259_g2 | AGCTAGCAGTCAAGGAACCA | TGGTTGTTTCTCTCCCTTGC | 130 |
| c24603_g2 | TTGGTCCTCTTTCTCGCCTT | TCCCTTTAGATGGCTCGTGG | 121 |
| c27711_g1 | GGGTGTCCTCATCAATCCCA | AGGCTTAGACCACTTGTGGG | 121 |
| c20784_g2 | GCCAGAATTGCACTAGAGCC | ATTGCTTTGTTGTCGCCCAT | 126 |
| c23128_g1 | AGTGACTGAGGTTCCCCAAC | CGAGCCCTTCCTCTACGATT | 126 |
| c15299_g3 | TGAGGGACAACAGCATCGAT | AGTAGTCGGTGCCACAGAAA | 110 |
| c28317_g1 | AATTCAGATCACCAGCCCCA | TTCTCTCCCTGGCATGGAAG | 110 |
| c23351_g4 | GTTGCAAGTCTCCCTCTCCT | TGTCCCAGTTCCACCATCAT | 130 |
| c30107_g1 | TTGCTGTTTTCGATGACCCC | AAACGTAAACCCAAGCCACG | 125 |
| c19657_g1 | ATGGCCGTTAGTAGGAGCAG | GTTGTGAACGGCATTGGGAC | 117 |
| c25215_g1 | GCAACCCACCATCTGATCAC | CTCTCCAGGTAGTAAGCGCA | 110 |
| c21688_g1 | ACCCGAGATTGTACCCAGAC | CCTCTCCACCTTTCTGCTCA | 126 |
| c21170_g1 | TGCTCCTTCCCCTGTCAATT | ATCACTCCATCCAAAGGCCA | 115 |
| c11322_g1 | TGGGGCGAATGGAGTCAATA | AAGGCGGTGAATTTGTAGGC | 115 |
| c19353_g2 | TGTAAGCCAAGTCACCGTGA | TTTCCAGATGCCCCTTCACT | 122 |
| c22194_g2 | GAAACCAGGCCAAGCATACA | CCATTTCATGAGGCTCGCAA | 123 |
| c15546_g1 | CAATTCCCTCCGGTCCCATA | AGAGAGAGATGGGGAGAGCT | 121 |
| c27711_g2 | CAAAACCCTGGTCCCAACAG | CAGTTCCCCTAGATTCCGGG | 128 |
| c18963_g1 | CATTGACGGTGATTTGGCCA | GCTAGACCGGGAGAGATCAC | 121 |
| c20259_g1 | TCAACTACTATCTGAGCGCGA | AATGCTAGTGATGGTGCCCT | 116 |
| c18021_g1 | CTGACTGATCTGGCTCTCGT | CGGTTGTTGCTGATGTCGAA | 110 |
| c19919_g1 | TGGGCAGAAAGTGGAAAACG | CTCCTCCATCTCCACCGTAC | 130 |
| c25380_g6 | TTGATTGCGGAGACAACACG | TGCCCATCTTCTCAGCAAGA | 120 |
| c28642_g4 | CCGGATTCTGCCCATCTTTC | TCCAAGCTTCAACCTTTGCC | 128 |
| c24292_g2 | TGGGTTGAGACTTATGCGGA | GCACTTGGGATTATGGCCAG | 111 |
| c28204_g2 | TGCTCTAGTTGCTCCATCAGT | CGTCTGTTGGGCAATGACTT | 128 |
| c315_g1 | CTGTAGGAGCCATGGTAGCA | CCATCCAGCAACACCAACAA | 116 |
| c20620_g4 | TCGGTTTCAACTTTGGCTGG | GCTTGAGGGCATGAGAACTG | 130 |
| c20414_g1 | AGATCAACTGGGGAGGCAAG | GTCTGGGAGTCGAGTGGTTT | 119 |
| c14854_g1 | TGAGGAGCCTGTCACTAAGC | GGTAGGATGGGTCGAAGGAG | 112 |
| c20982_g1 | AGACTGTCAAACTACGCGGA | AGTCCACCACAGCAGTACAA | 126 |
| c26708_g4 | TGGCCGAGACACCAACTTAT | AACAGCGCTTTCCTCCTAGT | 127 |
| c25213_g3 | GGGGACTGAGTGAGAGGTTT | CCCCTCAACAAGCTCTGGTA | 115 |
| c25215_g2 | TGGAGTCCAAGTGAGAGCAA | T TGACGACCTGATCATGGCT | 122 |
| c24463_g2 | TGGAGTCCAAGTGAGAGCAA | TTGACGACCTGATCATGGCT | 122 |
| c24857_g3 | AGGAGCTCATGATTAGGCCA | GGAGGCCCTTCATTCTTTAGC | 121 |
| c17716_g1 | TTGTTTCCACGGCAAAGAGG | GCTGCCTCCTCATCTTCCAA | 128 |
| c27912_g2 | CTCCGGATTTTGCCTGTGAG | ATTCAGCAGCCATGAATGCC | 111 |
| c17660_g1 | ATGCTGGAATCGGTAGCGTA | CGAAGCGAAGGACAGGATTG | 117 |
| c23362_g1 | AGAAGAGGGAGTAGGCATGC | CTCCTCCCAGACAAGAGCAA | 125 |
| c18087_g2 | GCAAGGAAGGCACAGGTTTA | CTTACTGGTTGCCTCGGTTG | 125 |
| c16755_g1 | CTGTCTCTCTGCGTACGAGT | CGATGCTCAGGTTGCTTTGA | 121 |
| c28757_g9 | GCGAGAACTTCCGGTCAATC | CATGATGGGGCAAGCGATAC | 114 |
| c20554_g1 | TTTCATTCCCTGTCTCGGGT | TCTTCTGCAAGTGGTCGTCT | 116 |
| R16m | CATGCAAGTCGAACGGA | CTTAACCCCAATCATCGAC | 716 |
| RhGAPDH | CTTAACCCCAATCATCGAC | CAAGGTCAAGGATGAGAAGA | 126 |
| RC001958 | CTTCTCTAACCGTGGCAAGC | GCTCAAGCTCCTTCGTGTTT | 136 |
| RC001958 | GGGGAGAGTTGAGCTGAAGA | AATACTCCTGCTGGCTGCTC | 129 |
| RC000216 | GCCCTAACACCACAACGAAG | TGTCAAGCTCGTCCAAACAC | 177 |
