# Supplementary material for: The Rosa chinensis cv. Viridiflora Phyllody Phenotype Is Associated with Misexpression of Flower Organ Identity Genes
Source: Front Plant Sci. 2016 Jul 12;7:996. doi: 10.3389/fpls.2016.00996 (PMC4941542; doi:10.3389/fpls.2016.00996)
Supplement: Supplementary file 4 [file Data_Sheet_1.DOC]

>c16755_g1_i1

TCTGAAGTTCGGACATAGACTCATGCATGAGTTGGTTCTTTCTTGATCGAATTTGCTTAAGAGAATTGTCAAGCTGTTGCTCCAAACTTTGGATCTCTTTGATACTCAATGAATCCAGATCTTCTCCCAAATAGTGCCTAAGGTTTCTTTGCAGAAGCTCAAATGGGAAGGGGTAGGGTTCAGCTGAAGAGGATAGAGAACAAGATCAACAGGCAGGTCACCTTTTCGAAACGAAGAACTGGGTTGCTGAAGAAGGCGCATGAGATCTCTGTCTTGTGCGATGCTCAGGTTGCTTTGATTGTCTTCTCTAACAAAGGGAAGCTCTTTGAATACGCAACTGATTCACGCATGGAGAATATACTTGAACGCTATGAGCGGTACTCGTACGCAGAGAGACAGCTAGTCGAACCTGATTTGGAATCACAGGGTAACTGGACCTTCGAACATGCTAGACTGAAGGTGAAAGTTGAGCTTCTGCAAAGAAACCTTAGGCACTATTTGGGAGAAGATCTGGATTCATTGAGTATCAAAGAGATCCAAAGTTTGGAGCAACAGCTTGACAATTCTCTTAAGCAAATTCGATCAAGAAAGAACCAACTCATGCATGAGTCTATGTCCGAACTTCAGAGAAAGGAGAAGGCAGTGCAGGAGCAAAATAACTTGCTTTCAAAGAAGACTAAGGAGAAGGAGAAGAATGTAGCGGAAGCTCAGGAGGTTCATGATTGGGAGCAGCAGCAACAGCAGCAAAACCATGGCCTTAACCTGGCAGCACAGGCTCCACTTCCATGTTTGAACATGGGGGGCACTCAGTCAGAGGAAAAGAGCATGTGCAGAAGGTATATATGGAAAAGGAAAATGGGAGCTTAAAAGCGGTTTCATAGAG

>c20784_g2_i1

CCTATATATACCTCTCTTCATCCTTTTATCTCCTACCTACCTACCCATTCCTTCTCTGCCTCACTTTCTCATTTTCAATGGCCAAGAATTCTTCTTCTTTCCCAACATACTTCATAGCCTTATTCATCATGAGCCGCCTGATCCCCACCATTGGATACCGCCTCCGCGACGACCCCGAAGCCATTATATTAGCTTCCACTGATTACGGCCACATCCACAGCGAAAACCCAGCCGCCGTTCTCTACCCTTCTTCCATAAACGACATTTCGTCCCTCATCAAGTTCGCCAACAATGTTTCCGTGCCGTTTGGCGTGGCTGCGAAAGGCCAGGGCCACTCGACACGGGGACAGGCCATGGCTCGCAATGGGGTCGTGGTCGAAATGTCGTCCCTGCGAAACCACCGACCACAACACGGCTCCTCCGGAATCGAAGTAGTTTCAACCACCAACAAAGACGAAGTGAAGCTTTACTACGCGGACGTCGGAGGCGAGCAGCTCTGGGTCGATGTTCTGCATGCCACATTAGAACATGGACTTTCACCGGTGACGTGGACGGACTACCTTTACTTGACCGTAGGAGGGACGCTGTCGAACGCCGGAATCGGCGGACAGACGTTTCGGTTCGGCCCTCAGATCAGCAATGTCTACGAAATGGATGTTGTCACTGGAGAAGGGGATTTTGTGACTTGCTCCCCAAACAACAACGCAGAGCTTTTCTATGGGGTTCTTGGAGGGCTAGGTCAGTTTGGGGTTATATCCAGAGCCAGAATTGCACTAGAGCCAGCACCAAAAAGGGTCAAGTGGGTGCGAATGCTTTACAGTGACTTCTCTGCGTTTTCCAGAGACCAAGAACGTTTAATCTCAATCAATGGGCGACAACAAAGCAATGCCCTTGATTATTTGGAAGGCTCAATACTAATTAACCAGGGTCCCCCGGATAATTGGAGGGCTTCCTCATTTTTTCCTCAATCCTCTCACAACAGAATTATTTCCAAAGTAAACAAACATGGGATCATATACTGCCTCGAAGTGGTCAAATATTATGACCATCACACTGAAACCACTGCGGATAAGGAGCTCCAAAATCTATTGAAAGGATTGAACTATCTTCCTGGATTTAAGTTTGAAAATGATGCGTCGTATGTCGAATTTCTGAATAGAGTTCGAAGCGGAGAGCTAAAGCTTCAGTCAGAAGGACTATGGGATGTCCCTCATCCGTGGCTTAATCTCTTTATTCCAAAAAGTCGCATCACGGACTTTGATCTTGGTGTGTTCAAGGACATTGTTCTGAAGAGAAATATTACCACCGGACCTGTCC

>c26880_g6_i1

TTCGTTTTTGTACTGCTTGCTGAACTTAATGAAAGGGCTGACCTATTTCGATCAAAAAAATATATTTATTGATCATGTAAATGAAATATTCTTTCCTGGAAAAAAAAATTGTTCTTGTTCTTTTTAATTTGTTATTTAGTTTCTTTTTAATCTCGAGTTAAATTCAAGTTTTGTTCAAGTCACACATCTCATATGTAGATTAAATCCTGTGTATTAGCTTAAGTATGTGAAAACTAGCCTTATGTATATACTGGAATGATAAACTGCAAAAATGTTTACCCCTATAGATTTGTGTTGGGTAAGTACAATGTTAAAAAAGTAACCAGCATTAACAAACAATAATTCATCCTGTCACTAAACTCAATATATATAATCTATGAAAAGACCTCCGAGCATAGATCTCAAATGAGTCAATTAGGTCCTGTTTTGACACGGCTTTTCCTACTCTTAATTTCTCTTATTTGTTAGTTATTTGTTGGGGTATGACTCATCTTCACATGTATATAACTTGTCATATATACTACATAACAAGGCTTCTGGTATCTCTTGTTTTCTCTTCATACATAGTATTTTGATGCTCTTGCTAAGTTTCTAAATGTATAAAATTTCTGATATTTCTTAATGTTAATTCTTTTACAAGCAGCATGCTCAAAACACTTGAGAGGTACCAGAAGTGCAACTATGGAGCACCGGAGACAAATGTATCTACAAGGGAGGCCTTGGAATTGAGTAGCCAGCAGGAGTATTTGAAGCTCAAGGCACGTTATGAAGCCCTACAAAGAAACCAAAGGAATCTTCTTGGAGAAGATCTTGGCCCTTTAAGTAGCAAAGAGCTTGAATCACTTGAAAGGCAGCTGGA

>c23351_g4_i1

AAATAGTTCCATGATAGCATGGTCGCACAAGGAGTTTGAAAAGGAGGGTGTGGCTTGGGACATTTGAGAGTGCAGAAGAGGCAGCACGAGCCTACGACGAGGCTGCGATTCTGATGAGCGGTCGAAATGCCAAAACAAACTTTCCAGTCACTACAAATGAAATTGCAGCTGCTAGTAAGGATCCTATCAACAAGAGCAACTCCAGTAGTCCTTCTTCACTTTCTGCAGTCCTCAGCGCCAAGCTTCGCAAGTGTTGCAAGTCTCCCTCTCCTTCCCTCACTTGCCTCAGGCTCGACACTGAGAATTCTCATATTGGCGTTTGGCAAAAGCGTGCCGGGCCACGTTCAGATTCGAATTGGTTGATGATGGTGGAACTGGGACAGAAGAGTGATTTAAAAGAGGAGAAGGAGGAGGGTACTAGCGCCCCTGAATTGCCCAATGGACACGAAACGGAAGGAGTACACGGTGGGGATGATAGATTGCACGAGGAGGAAAGGATTGCATTGCAGATGATAGAGGAACTTCTCAATAGAAATTAATCACTTGGTATTTTATTTTTTGCGGTTCCGACGGATGATACGGAATGAAGGTAAATGTTTTATCGTGATCTTTAGACTTCTCTGTCACTCGAGCTAGCTACAAATCTATCGTAATCTTTAAAATTAGGTAATCGTACGTATAGGAGTCCTAGTCAAGTATCAACATCACATGGAGGTTGTTCATACCACCAAATTGTATGACGAATTGCCATGTGCTTAGTGTTATCAATGCATTGTCTAAATATGTATGGTTGAAGGAT

>c17660_g1_i1

ATGGTACAGTACCTCAAAATTTGATCAAAACATGGTTAGATTATTGCAATTTCATTTTGCAAGAAACATTCTTGTTCTTATTCCTTTACATGGTACAGAGTAGAGACTAGAGAGGTAAATTAAATTTAGTGATGCCTTGATGAATCTTGCAGTTGATGAACATCCCAAATTAAACAATAGGATATATATATATTTCTGATGTAATCTGCATGCATCATTTATTCAAATGGCGAACCATCCATGGTGGCATGAGTGTGTTACTGGCAGGCCGAGTTTGAGCGCTTCCCTGATGATCCTCAGCATCTTCATCCATTGCGTCTCCTCTTCCCTGGAAAGCCCCACCAATAGTTAGAGAAGAGAGTAGGGCTGGTGGTGATGTAGCTTGCAGTGGCGGCATTAAGACGAGGGTTGAGGGGTTTGGCTGCTGATTATTATTAGGTTCTTCCTGCAGAAGCTTTTCATTCTCCTTCAGCTTCTTTGCTAGCGAGTTGTTTTGCTCTTGCAGTGTCCTGTGCTTTTTCTGCATCTCTGAAATGGATTCATGCATTACTTGGTTCTTTCTTGCTCGCACGCGCTTAAGAGCTGTATCAATCTGTTGCTCCAAACTTTGAAGCTCCCTCAAGCTTAAGGGGTCTAAATCCTCTCCCGTGTAATTCCTTATTTTCCTTTGTAAGATTTCAATCCTTGCCGTAAGCTTGGGAAATTCCATGGACCAGTTTCCCTGTGATTCAGAAGCAGGAACGCCCATGCTCTGCCTTTCTGCATACGAGTATTGTTCATATCGCTCCAGGATCCCCTCCATGCTGGAATCGGTAGCGTACTCAAAGAGCTTGCCTTTGGTGGAGAAGACAATCAAAGCTACCTCAGCATCACACAGAACTGAGATCTCATGAGCTTTCTTGAGCAATCCTGTCCTTCGCTTCGAGAATGTTACTTGTCTGCTGATCTTGTTCTCGATCCGCTTCAGCTGAACCTTGCCTCTTCCCATTTTTGAGGAATTGAGTTAACTTTCTTTAGAATTTAACAAAAGAAAGGGGGCTGTAAACGGATATCGATCAGTTCCTAAAAAGAAATTGATCACCAGAATAGCAAAAAACTGGAAAACCCTAGTTTGGTTCGTTCAGGAAAAGAAGATGAAAGGGGAAAGGAGGATGATGGTGAGGTTCTTGTTCACCTAGTAATGGCAGTGTTAATGAGGCTTTCTTTGGGTATCTTTCTAGTTTTGTGTTTGGGTGGAGTTTTTGGAAACAAAGGCTCATGTATATGGGAAAAGAAACTAGGAGAGAGAGAGAGAGAG

>c20760_g2_i1

AACTCCTAAATCATCATCATCACTCTCTTTTGCAGATGGACAACTTCTTATAGTTTCTGGTAATTAATCAATGAATTAAAAGCTGGTTGTATCCGAAACAGGGGTGAGGAGATAATGGTGACAAAGGAGGCACACTGAAAATTCCGTCAAGATCTTTGCTATCCCAAATTGACGTCCAATTCGACGGCGTTAATGGACAGTTAACTGCTGCGGCTGATGAAATGGATGATCTAATTGGTGAGGTGCTGTTGCAGCTGCTTATGGTTTCCTCCTCCTTCTTCATCGCTTTATTACTCTCCACACCCTCACTGTTACCCTCTGCACTCTCAGACCTCCTCCTCTTCCGGCCAACTACGCTCGCCTGAAGAGGCTGAGCCGCCGCGATGTTGCTGTTAATTTCAAGAGGAAAGTTGAGGATGGCTTTGCTCCCCCTCAACTTAAACGCCGCGAGATCATAAGCCTTGGCCGCTTCAACGGCGGTGTCAAAGGTCCCCAGCCAAACCCGTGACCCCCTTTTGTTAGGGTCACGAATTTCGGCAGCGTACTTGCCCCAGGGGCGTCTACGGACTCCCCTGTAGTGCCTCGCCTCGTTCTCAGAGTTTATCGAACTCTGAGCAGTTGTGACGCTCGCAGAAACAGCAGGCGCCACAGCTGGAGCCGGCCTTGTTGGTATTGCCACGTTGATGGTCGGCTTGCGGTGACTGAGAGTCGAATTTTTCTTTGGGGATGGCTCGGAGTGGACAATCTGAGGCTTGGTTTCGAACTCGAATTCGGGTTCAGAAGGGAGTAAGTAATCAGAGACTTTGAAGGAATCATCAGCTTGTTGATATTGAGGGGTTTGATCAATGTTGGAGGTGCAGTTGTTGAGGCTGGTGATGAAGCTCTCCATGGAGTTAAACTCACTGAGAAGGTGTTGGCTGATCATGTCTAGGGCAGAAGCTTGTTCCATTTTTTATGTTTGGAAGTCTCGGAAGGAATTTGAAGTTTGAAATTCAAAGTGAAGAGTAGTGGTTTGAGTGGAAAAGAGGAGTGTGAGGGTGGTTATATATATGCGAAGGGGGGAGGAAGATGGGAAGGAGTCGCAAATTGGGGAAAGGTCTGAATGTGTGGCTTCACATTAGGTTCGAGGACAGAGATAGGAAGCATCATTTTGGATTTTTG

>c18087_g2_i1

AGCATCTTGAAGTTTCGAAACGGTTCACAACCTTGAAACTATTGGGAGAGAGTCTAGGACTATGCAGCATTGACGAACTGCAAGAAATAGAGCAACAGTTGGAGAGGAGTGTGAACAGCATTCGAGCAAGGAAGGCACAGGTTTACAAGGAACAGATTGAGCAATTGAGAGAAAAGGAAAGAGTCCTCACAGCTGAAAATCAGAGGCTAAATGAGAAGTGTGAGGCTATGCAACCGAGGCAACCAGTAAGTGAGCAGAGAGAAAACTTAGCCTGC

>c20518_g1_i1

AAGCCAGGTATCAAATTGGTTCATTAATGCAAGGGTGAGGCTTTGGAAACCCATGGTTGAAGAAATGTACGTGGAAGAAACAAAGGAGCATGAAAACAACAACAACATGTCCTCCTCAGATGGTGTCACTACTGATCATCATGGCGATCCGAGCACGCAGATGACACGACCCGAAGATCAGAAACCGACCCAGGACCAACTCCAACTCGTCCGAATAGACTCGGAGTGCCTCTCGTCCATCATCACAAACAACCCAGGTGAGAAAAGTACTGGCCATGACTCCAGAAGCGGCAAAAGCGCCCTCCAAATGCACCCACAGCACAATTTCGGGCGGGTAACAGATGCATTCGGATCATCGATGGAGCTGGATTTTTCAACTTACAATCATCATCACCAGTCTGGAGGCGCAGTTACTTATGGTAATGAAAACCCGAACCAGAGCTTCAATGGGGGAGGGCATGGTGTGTCATTGACATTAGGGTTGAACCAGCATGGTGGTACTAATGGGGTGAGCTTAGCATTCTCTCCTTCCTCACAGAGCTCTCTCTTCTACCCTAGAGATCACATCGAAGAGTGTCAGCCGGTTCAGTACTCGTCTCTATTAGACGGCGAAGGCCAGAACAATTTGCCTTACAGAAACTTGATGGGTGCTCAGTTGCTCCATGACTTGGCTGGTTAGGTAAAAGTACAATATTGCTCTTGGATGATCAGGAGGTTCGATCACTGTGGTTTCAATTGGAGGTGATAATAGATAAATAGTGGGTAGGAATAGATGAAAGTTAATACTTGGGATACATACATAAGGGGCTATACTGGGTTAGACAAGCAGATTAATATGATCAACGTTTTCATGGCTGCTGGTAGTTAATTAATTAATTTTGGGTACTCCTTTTTCATTTTCATTTATAATTTGTATGAACGTGGTTTTATTATTGCTAGGCTAATTTGGAATTGCCAGTGATCTGGTGCAAAGATGATTCAATATTTGTTTCCTCTTATAT

>c27740_g2_i1

GATAGCAGATGATCATCAGAATGCCAACAAGCAAAACAAAATTCGAAGCAACAGAAAGCCCATATCTTAAGCATATAGCTTGTGGAATAATCTTAGAAGCCGTAGTGAAGAAACAGCAGGGAGATTTGGTGAAGTAGCACTTATCTTATTTAGCACCTCAAAACTTTCTCAGTGTGTGGGTGATTCTTCTTCTTCTTCTTCTTCAGAAATTCAAATTGCAACTCAGAAACATGGATATAACAAGCAGCACAACAGATGAAAGCACAAGCAGTGAGCTCTCAATGTCAATTTCACCGCCTCCGAGACCAAACAACAAGCTCTCCCCTTCGCCGCCGCAGAGCCTCTGCCGCGTCGGCAGCGGCGCCAGCAGCGTCATTCTCGACTCCGAGAACGGAATCGAGGCCGAGTCCCGCAAGCTCCCGTCCTCCAAGTACAAAGGCGTGGTCCCCCAGCCCAACGGCCGCTGGGGCGCTCAGATTTACGAGAAGCACCAGCGCGTCTGGCTGGGCACCTTCAACGGGGAGGACGAGGCCGCCCGGGCATACGACGTCGCGGCGCAGCGCTTTCGCGGCCGCGACGCCGTAACTAACTTCAAACCCCTAGCAGAGTCTACCGACAACGACGACGACGAAATCGAGGCAGCTTTCCTCAACTCCCACTCCAAGTCCGAAATCGTCGACATGCTGAGGAAGCACACTTACAATGACGAGCTGGAGCAGAGCAAGCGCAACATGGGATTCGACGCCAACGGCCAGCGGCGGGCACGGGGCGAGAGCGGCCCAATGGGCCCGGACAGAGCCGCTAAGGCGCGTGAACAGCTTTTCGAGAAAGCTGTCACGCCCAGCGACGTGGGCAAGCTGAACCGGCTGGTCATCCCGAAGCAACACGCCGAGAAGCACTTTCCGCTGCAGAACGGAAGCACGTGCAAGGGCGTGTTGCTAAACTTCGAGGACGTCGGCGGCAAAGTGTGGCGCTTCCGGTACTCCTACTGGAACAGCAGCCAGAGCTACGTGTTGACCAAAGGGTGGAGCCGCTTCGTGAAGGAGAAGAATCTCAAGGCCGGCGACATTGTGAGTTTCCAGAGGTCGACCGGGCCGGACAAGCAACTTCATATAGATTGGAAGGCAAGGAGCACCGGACCGAACCCGGTTCAGTCCGGGCCGGTTCACATGGTCCGGCTATTCGGGGTCAACATTTTCAAAATACCTGGGAGTGGCTCCCCTGGTGTGGACGCTCCTATTGGCTGCAATGCTTTGGGGAAGAGAATGAGGGAGATTGAATTGTTGGAATTAGAATTTAGCAAGAAGCCAAGGATAATTGGAGCTTTGTAACATTTTTCTTCTTCTTCTTTTTTTTTTTAAAAAAAATTATTGTAATTTTTTTTCCTTAGTAGTTTGTGAAGGGGAAGGAGTTGGAGGACGGAGATGGGGAGGTGTAGTTGCAAGTTGCAAAAGGTGAAGTTGTATATTACATGACCATAAAAAAGCTGAAGAAAGCAAAAGCTTTCATTTGAAGTTTTTACAAAATTTTCTCCAAAGGAGGATTTTAACTGTCAATTTGATATTGTTCAACCTGTTTCTCTTGTTGGTGTAGGTGTTCTTATGTTATGGTTTTCCAAAACATTGTAGATTATTTCTCTTTGTTCACCAACTGATGTATGTTTGAGAAGGGAATGTATGGAAGGTTTTCTAAAACATTATAACTTTTGCTCCTGCTAAAAAAGTTAGGCCTATAAAAGTTTGGCTTCATTTGTCAAACGCTAATAATGAACATTTTATCG

>c24715_g4_i1

CCTATTTATATGCAAAGCTTAAAAGTTAAAACAAACCTCTTGTGTGATATATGGTATAGCTTTTTGGACATATGGTAGCAAGCTATGGAGTTAGAGGAATATAGTGGAGCTGGTTCTGTAAAATCTTCAGATATAAGTGGTGATGGTGGTGGCGGAAGAAGAGATGGTGAAGGTGGCAATAATGGCTTGATGTATTTGGTGTGGGAAGATGTGAGTGTTGTGCTTCCAAACTTTGGGATTGGAAATAGCACTAATGGAGGACACACCAGAAGATTGCTTGATGGCCTCACTGGTTTTGCTGAGCCTGGTAGGATCATGGCTATTATGGGTCCTTCTGGCTCTGGAAAATCAACCCTTCTTGATGCTTTAGCAGGTAGACTGTCACGAAATGTTATCATGACTGGAAATGTTCTTCTTAATGGGAAGAAGACGAGACTTGACTATGGTGTTGCTGCTTATGTGACCCAAGAAAATGTGCTGTTGGGAACCCTAACAGTACGAGAAACCATAACTTACTCGGCTCATCTAAGGCTCCCAACTAGTCTGACCAAACAAGAGGTAAATGACATTGTTGATGCCACAATTACCGATATGGGTCTGCAAGACTGTGCTCACAGGCTTATCGGAAACTGGCATTTGAGGGGCATCAGTGGAGGAGAGAAGAAGAGGCTGAGCATTGCACTCGAAATACTCACAAAGCCAAGGTTGTTGTTTCTCGATGAGCCTACCAGTGGCCTCGACAGTGCTGCAGCTTTCTTCGTAGTTCAAACCCTCAGATACATTGCTAAAGATGGCAGAACTGTCATTTCCTCGATTCATCAGCCCAGTAGTGAAGTGTTCGCTTTGTTTGATGATCTTGTTCTGCTTTCTAGTGGCCAAACAGTTTATTCTGGTCAAGCAAAAATGGCAGTAGAGTTCTTTGCTAAAGCTGGAGTTCCATGCCCAAGTCGAAGAAACCCTTCTGATCACTTTCTTCGTTGTATTAATTCAGACTTCGACGAAGTCACAATGAACACCTCTCACAAAATACGTGAAATCCCAAAATCATCAGAACCTTTAATGAACTTTGCAACAGCAGAGATGAAAGCAATGCTTGTTGATCAGTACAGACGCTCGGAGTATGCAACAAGTACAAGAATCAGGATTCGAGAAATATCGTCCATGGAAGGACTTGGGGTAGAAAAGAGGAGTGGAAGCCAAGCAAAGTGGTGGAAGCAACTCTCAATATTGACACAGAGGTCTTTCCTCAACATGTCTAGGGATATGGGGTATTACTGGGTGAGAATCATCGTCTACATACTTTTATCTCTTTGCGTTGGCACAATTTTCTATGACCTGGGAACAAATTACACTTCAATATTTGCAAGAGGAGCTTGTGCTGGATTTATTTCTGGCTTCATGACTTTCATGTCCATTGGAGGCTTCCCATCCTTCCTTGAAGAAATGAAGGTTTTTCATAGAGAAAGGCTCAATGGGCATTATGGAGTTGCTGTCTTTACTCTGTCAAATTTCCTCTCTTCCTTCCCATTCTTGGCTTTGATGTCAATTGCATCCTCAACTATAACTTACTACATGGTGGAAAAGCATTCTCATTTCTCCAACTATGTGTTCATGTGCCTTGATCTTCTCAGCGCCATTGCAGCGGTAGAAAGCTCCATGATGATCATAGCTTCTCTTGTTCCGAACTACTTAATGGGAGTAATAATTGGAGCAGGATACTTGGGAATCATGATGATGACTGCCGGATTCTTCCGCTTCATGCCTGACCTTCCCAAACCAATTTGGCGCTATCCAGTTTCATATCTGAACTATGGTGCATGGGCGCTGCAGGGAGAATACAAGAACGGATTGATTGGCCGTGAGTTTGATTCTCTTACACCCAATGGCCCGAAGCTGAAAGGCGAGGACATGCTCAAAACTCTGCTGGGTATTCAACCAGATCAATCAAAGTGGTGGGATTTGGCTGCTGTCTGTGCCATTGTTGTATCTTTCAGATTTATTTTCTTCATCATCCTTAAGATCAAAGAGAGAGCTTCACCTCTTTTCCGAGCATGTTATGCTAAGCAAACTCTGAAGCATCTCAAGAAGCGACCCTCATTCAGGAAAGATGTAACTCTT

>c20982_g1_i1

TTCTCAGGCACGTCCGCATGTGTCAGGAATCTAAACTACTATTTCTCCAACTCTGATGAGCTTAAAAGGTCTCACACATTGTGCTCTTGAAATCCCAGAAGATATCAGTTCTCAAATTTCCAACACTAATTTGCAAGACACATACTCATGAGTTTCATAATCACCTTCATCTTGTTGCTATCAACCACACTATTTCATCAATCTTGGGCTTTGGAGGAGGTAGCTTATGGATCAGAGGCTTCAGAGCTTGAGGTTTTGACTAGGGAGCTATTGGAATTGGCGAGAGAGCCTGAGTTCTTTGAATGGATGAGAGGGCTCAGGAGGAGGATTCATGAGTATCCAGAGCTGGGATTTGAAGAGCACAAAACTAGTGAACTCATCAGGGCTGAGCTTGACTTACTCGGGATTGAGTACAAGTGGCCTGTGGCCAAGACTGGTGTGGTGGCCTTTATTGGGTCTGGCTCTAAACCAGTCTTTGGACTTAGAGCTGACATGGATGCTCTCCCTCTCCAGGTTCTCTCTCACACACTTTTTTACTGTGTCATTCCAAACAATGCTCATGGTGCCTCTGTTTTCATCTTTCTCAAATGTATGACGGTAATATTAGGGTTAAATGTTTGTTGATATGCTTTATAGTTGTCACTGTTCTACGGCTACATGGACTTGGTCTGGACTATGTATTTGATGAGTTGAAGCACTTAAGCAGTCCCAGTTAATTAGTGTCTCACTTTTGCATTCTATATAAAGAGCTGATACGGTTAAAATGGAGCCTCGTGTGATTGCAGCTCATAAGTAAATCACTAAATCCTATAGATAGTGCTAAAGTTTTCACATATTGTTTGACAACGATTGATGGATGATAGATATCCTTTAACTAGTTAAACTTATTGAACTGATGCCATGAAATCACTCTATCCTTGCTATTAATAAGGAACTAGTAGAGTGGGAACACAAGAGCAAGATTGATGGAAAGATGCATGCTTGCGGTCACGATTCTCATGTAGCAATGCTACTTGGAGCAGCCAAGTTGCTTCAACACAAAAGAGACATGTTGAAGGTAGATCAGAAGCTGTATATATTTTGTTAGCTACTTTGTGGGTGCTAATATTTCAATAATGTAATTTAAGGGAACTGTGAAACTAGTGTTCCAGCCTGGAGAAGAGGGTTACGCTGGTGCTTACCATATGTTAAAAGATGATGTTTTGCATGATATCGATGCCATCTTGAGTATACATGTTTTTCCATTAGTACCTACTGGTGCCATAGCTTCAAGGCCTGGTCCGATACTTGCTGGTACCGGGCTCTTCTCGGCTACAATACAAGGAAAAGGAGGGCATGGAGCAGCTCCACATGAGACTAGGGACCCTATTATTGCAGCAGCCTTTTCAATCATTGCTCTCCAACAAATCGTGTCTCGGGATACAGATCCTCTTGACTCAAGAGTAAATCTTACTTGCTTCCTTTTTTCTGTATCATTTTAGAGGCAATCATTATCATACTAATCTTACAAAAGACTAGTGGCCAAAAAAGAAAAGAAAAAAAATAGATTATCTGCCCAAGACACAAGCTGAGTAACAAGTAGAATGAGGGTCTGATCCATTCATTTTGTGTTATGTAGGTGGTAACAGTTGGGCACATACATGGAGGTCAAGCAGGAAATGTTATCCCAGAGACTGTCAAACTACGCGGAACTTTTAGGAGCTTCAGTTCTGAGGGTCTTTCCTATCTCCAACAAAGGATCAAAGAGGTCATAGAGCAGCAAGCAGCTGTGCATCGTTGTACTGCTGTGGTGGACTTCATGGAGGAAAAACCTATGCCTAATCCAGCAATGGTTAATGATGAAGCAATATACGAACACGTGAAGAAAGTCGGTGAGGTCCTTGTTGGGAAACC

>c17791_g1_i1

CTTCTGGAAAAAAGAGCTAGCTTGCTAGGGTTTGCTCATTTTTGGTAAAGTCATGGTTTTTAGGATATTATGTATGAGAAGTTGAAGAAAAAATATTTTTGAAATGTCTGAGATTTCATCAAAGTGTTTTACCTAAAATTGCGCGCCCTTTCCTGGAAAGAGGATCCCAGTAGTATGACCCAAACCCTTGAGAGATACCAACGAAGCAGCTTCAGTCCTCATGACAACTCCACCAAACGGGAAACAGAGTGCTGGTATGAAGAAGTCTCAAAACTGAGAGCTAAATATGACTCACTTCTGCGTACTCAAAGGCATTTGCTTGGGGAAGACCTTGGACCACTGAGTGTGAAGGAGTTGCAAAATCTTGAAAAACAGCTTGAAGGAGCTCTTACACAAGCCAGACAAAGGAAGACACAGATTATGATTGAACAAATGGAACACCTGCGCAAAAAGGAGCGACAGCTTGGAGACCTTAACCTGCAGCTTAGGGATAAGCTTCAGAAGGAGGGGGAAAATCTCAAAGCCATTCAAGACTTCTGGAGCTCCAATAGTGCAGCAACTGGGAATAGCAACTTCTTGCATTCTTCACAAGTCAGCCACATTGATCCCGCACCTGAACCCATCTTACAAATTGGGTACCATCACTATGTTGCTGCTGAATCCGCAGATGTTCCGAAGAGCATGGCCATGGCTGCCGAGACCAACTTCATCCAGGGATGGGTGCTTTGAGCACACTATGTCTAGAACTCTAGCTTCAATCTTCAATATAAATAAACTTTATATCAGGATTTGCAACTAAGCTTTAGCATATTTGATGTTTTGTACTGGAAACTGCATACAGTTGTCCAGCATATTCACATACTTGAAAATATGGATGACAAGATCAAGATGTTTACATTTGTTGAGCACTTGCTTGAATGGCAGTTATGTTTCTTTTTTCCTGTGTTTTCATGGAGCTCCGA

>c22194_g1_i1

ATTTTTTAGTCCATCAGTTCACAATTTCTTTTAGTCCAATTCATGTTTTTAGCTCCACAGCTAGATCAGCAACTGTACTTTGATGTTTGTTGTCAAAGAACCCAAAGTGCAATGAACTTATTAACTCTCAGTCCATCAGTTCACAATTTCTTGAGCTAGTTAGCTAGTTGTAGTGTAACACCATGCTTTTCTTGATCAAAGCTAGATAGGGGAGATTAGTACTATTTAGAAACCGTCTTTTGATTTTCTAATTTGTACATAAAGTTTGATCCTTTTTTGCTTGATTGATGATTCATTGTACATTGGCCTTTTTTTTTTCTTTCCCCTACTTGATTGATGACTCTCAGCTGCAACTATGGCCTATGAAAACAAACCCAACACGGTCCTGGACGCTGATGCCCAAAGAAGATTGGGAAGGGGAAAGATCGAGATCAAGCGGATCGAAAACACCACCAATCGTCAAGTCACCTTCTGCAAAAGGCGCAATGGTTTGCTCAAGAAGGCCTATGAGCTCTCTGTGCTCTGTGATGCTGAGGTTGCTCTCATAGTCTTCTCTAACCGTGGCCGCCTCTATGAGTATTCAAACAACAGCAGTGTTAGAGAAACAATTGAACGATACAAGAAGGCATGTGCAGATTCTTCAAATAATGGATCTGTCTCTGAAGCTACTACTCAGTACTATCAGCAAGAAGCTGCCAAACTGCGTGCCCAGATAACCACTTTGCAGAACAGTAACAGGGG

>c18211_g1_i1

GTTTAAAAAAGCTACACACGCAAAGAATCAACTTAATCTTTGTGTTCCTTTATTGTTATATGTTGTGATCACATTGAAGCCTTTTCTTTTGCCAACAGATAAGAACTAAGATATTCAATTTGGGGTAGTTTTTACCATTTTGATCATCTTTGCAAATTATCATGGAGTTCCCAAAACAAATTACACCAGCTGATGATCCTGAGAGCTCTTCCCAAAAGAAATTGGGAAGAGGGAAAATCGAGATCAAGAGGATCGAAAACACTACCAATCGACAAGTCACATTCTGCAAGCGTCGCAACGGTTTGCTTAAGAAAGCATATGAACTATCTGTTCTTTGTGATGCTGAAGTTGCTCTTATTGTTTTCTCTACCCGGGGGCGCCTCTATGAATATGCTAACAACAGTGTTAGAGCAACAATCGAAAGGTACAAGAAAGCGTGTGATTCTTCAAACACTGGATCTGTAACTGAGGCTAATGT

>c15299_g3_i1

GTCCAGCCTTGGGGAACGGGTGCAAAATAACCAAATTTATTGCTTTGTAAAACTGGCTAGGCTAGCTTTCACAATGGTTAATTAAGCACTTGGCTTCATATATGAACTGCTCCTTCAGTGTCAGGAGTTCTCAACATACATTGAAAACCAGTAAGAACAAAATAGAGAGTACAAATAAGCCTCATTTGGCATCTCATCTGAGGAAATGCATACACAACCTCACAGCAAAACCTGAAAACAACAAATGAACCCGCACACTAGAATTCTATATAGGATTCCACCAACTTAGATTGCAAGGAAATGGTAAAGTATGAGGGACAACAGCATCGATAATAGAGGGCATAGAAAAAGGGCTTGTTTATCATCCATATATTTTGTTTCGGATCATATTTCAGGAGCAATTTCTGTGGCACCGACTACTTTCTCATCATGGTCACCACCACCCTCGAGTTGGCTCAAGTTTCCTTTCTCCTTGATGAGTTGGGTGTGGTGGTGCTTGCAGTAGAGGCGGCCCTCATGTGCAATGTAGTTCGATGGGCTG

>c20414_g1_i1

ATATACTTCATCCGTCTCCACATCTGCATGCATTGTGACATTGTGGAGCTGACAGATCAACTGGGGAGGCAAGGCCGGGTAGTTTGGTATGTGAGCATCAACTTGTTTGTTAGTAGTAGCAGCAACCTGGTCACTGTGGCCCTGAGGAAAGTAAACCACTCGACTCCCAGACGTCGGCAGCGACACCAGAGGCCCTGCGCATGCATGCCATAGCTCCGAATTCAAGCACTTCTTCTCCGCTCCTC

>c28204_g2_i1

AGAAGTTGCTTAAATGTCAGCCTTAATTCTAAAAGGTTAAAGGTTCTACTTATTCTCCAACTAAGGAAAGGTAGCTACAATTTGCAAGAGCAATAACCAAAATACATTTTCCTTCTTCTAGCCTTAGAGTAAATTACTTGAGAAAACTAGTAAAACTAACAGACAAACTTAGTCTCCACCAACAGAGTCAACTTCTATGACCTAGGGATTCTACTCTCTTTTACAAGTTACTTAACTGCACTTTCAACGATCGCTGCTAAGCTAGACTCTCAACTGTATCAAATTCCAACTCCAGAACCTGTTGATTCTATGGCAAAGAGAAACTCTGGACCAATTACAGGCCTAGTTGGCTCCCTGAGTCATTAGAGTGGCTTTCTCTTTCTGGAGCCTTCCTCTTGCGATATGTATCGCTTGGCAGCCCTAATTGCAATGTAGTGTCAGAATCTCCATTGTCCAATGCGAAATTGCTGACCAAATCGGGGCTTTTGGCACCATGGTGCACAGGGGAGTTCTGTTTTTCTACAGGACAATAGTCAAGATAAGATGGAACTGCATGATCAGCTTGGGGAAATAAACGCCGAAGCTCCTCAACCTGTTTGCGCAAGGTTTCATTCTCGTGTATAGCACGCTGTTCCTTTACTCTTGATTGCTCTAGTTGCTCCATCAGTAATTTTTCCTTTTTCTCCTTCACTGAAAATAATCCTTCAGTTAATTGATTTTCTAGTTTCTGCAATTCTTTCAAGCTCAAACTAGACAAGTCATTGCCCAACAGACGCAATTGATTCTGTTGTAGCTTCTCAAGTTCATCTTTTAGAACATCCACGGCATCCTCCTCTGCCCTTGATTCTACCAGAGCGGTCTCTGAAGACTCATAACACTTGCTGTATCTTGCAATAGTTTGCTTCATACTGTAAAAAATTGGTACGGATAAACATCAACAGCTATAATTTGTATAAAACAGGGTACAATTAATTTCATGTGGCTATAGCACTTGCATTGCAGCATCATTTCTAAGTTATTATATATGAAATCAGTATTAGATCGGCAACCGAATTAATGCTTCGTCTCAAGCTTAATGCAATCATACAAGAGATGCTCTAACCGAAAATATATGGTAGAAAG

>c24463_g2_i1

GGTCCGATGAACAATCTAGTCTCTACCTGATCTGAATTACTAATTGTTTGACTGCTGCTACTTTGACTACTATTCTTCCAGTTACAACTTGCCGCCCCAACTCCTTTTTTTTGAATTAAAGGTTTCTGACAACGCCTTGCACCACACTCTGTGTGTAGTTTTATATTCTCTTCTAAGAGGAGCCTCCCCTTTGCTTTTAGTCGTTCTATCTGCTCCATGAATAGCTGAGCCTTTCTCTCTCGTACAATGCACAAGCTTCGCTCGAGCTGGCTACTGATCTGATTGAGTTCTGGAGCAGAGCAAGAATCTAAGTCATGTCCCAAAAGCCTCCTGAGAGATATAAATTTTAGACTTTAGGAATACATAGTCTAAATGGAATGTCAGATGTGTGTGCAAATGTACATGAAAATGAAGTTCAAACTCAGTCATAAACCGTTGAGAAGCTTCTAGGATCTCAATCTTCTTGGCCATGTCAGCTGATTCATGCTTCAATTGCTGCATACACTGTTCCACTTCAATAGTGTTGGTTTGCACACCTTTTGCATGTTTATGGTATTGGTTTATAGTCTTTTGCATGTCAGAGCTGGAAAACTCGTAGAGCCTGCCATTCTGAGAAAAGATGATCACTGCAACTTCGGCATCGCAAAGAACCGAAAGCTCATAAGCCTTCTTCAACAACCCGTTTCGACGTTTCGAAAAGGTTACCTGCCTGCTTGTCGCATTTTCGATTCTTTTCATCTGAATCTTCCCTCTCACCATCTCCTGGATAGTCAAAACCCAGTCCCCCAAGAAAGTATCAAACTTGGGTGTAATCAAAACACAAATCTCAGAGATGAAACCCAGAAGCAAAACCCCTAAAACTTGTCAACTTTAGGTTTCTAAAAACAGAAACCAAGGACCCAGTAAGCTAACAAGGACTGCTCAATTTACACAGATATGGAAGAGCAGATCAGAAACCCTAGAAATAGATGAGCAGTGAAGATGAAACAACTTTTTTATCGTTTTATCCGATAGTAGAAGTGTCGATGTTGTACCAACCTCTTTATTCTGAGGCCACACAAACCTTTATTTCCATATTCATCAATACCAGCTTCCATTATTCACCATGTCTTCTCTGCTTTGCAAGTGCCTTTCATTCTCATTGCTTATCTTGTTTTGGCGAAGATTTTTAAAAAACGGAAACTCTCAAC

>c21541_g1_i1

CTTATTTTCATCTAAAAAATACTCATGGTCCTTCCCATTCTCATTCATTACTTGGTAGTTAGTCCCCTACCGATTCCCACTCACCCTCCTCTGCCACCTATAAATAAAGCTGCTTCCCACTTCATCTCCATCATCGTCAACATTCAATATTCAACCCTCCTTTTCCCTCTTTAACCTTCCCCTCCTCTCAACTCTCATCTCTCACCAACAAAAGAAACAACTAGTCAAAGCCACCTGCAAATTAATGGCTTCTACTTCCTCCTTAGCTTTCCCTTCTCTGCAGCTACAATTCCAAACACCACGCAAGTCATTCCCATCCACTCGCCGTATTTTCCTCCGTCCGATCTGCGCATCCGTCTCGGAGAAGTCATCGTCTTCATCCTCCTCCATCTCATCACAACCCGCAGAGCCAACCAAGCTTCCCTTGAGGAAAATCCCCGGCGACTATGGCCTTCCCTTCGTGGGTCCCCTCAAGGATCGCCAGGACTACTTCTACAACCAAGGCCGGGAGGAGTTCTTCAAGTCTCGTATCCAGAAGCACCAGTCCACCGTGTTCAGAGTCAACATGCCACCTGGCCCCTTCATCACCACCAAATCCCAGGTCGTCGTCTTGCTCGACGGCAAGAGCTTCCCGGTCCTCTTTGACGTTTCCAAGGTCGAAAAGAAAGACCTCTTCACCGGCACCTACATGCCCTCATTGGAGCTCACCGGCGGTTACAGAATTCTCTCCTACCTCGACCCCTCGGAGCCCAAGCACGACAAGCTCAAGCGTGTAATGTTCTATCTCCTAAAGTCTAGTATTAAGTCTGTGATCCCTGAGTTCCACTCAAGCTACGCCGAGTTTTTTGAAACTCTGGAAACCAAGCTCGCTGATAATGGTAAAGCCAGCTTCAACGAGGCCAACGATCAAGCAGCTTTCAATTTCTTGGCTCGCTCACTCTACGGTGCTAATCCGGCCGATACCCAACTCGGCACCGACGGTCCTAAATTGGTCCAGAAATGGGTTCTATTCCAACTGAGTCCGATTCTAGTTCTTGGTCTACCAAAGTTCATTGAAGATCCTCTGTTTCACACCTTCCCTCTCCCACCGTTTTTGGTCAAGAAAGACTACCAGAGACTCTACGACTTCTTCTACCAGTCATCCGGCCACGTGCTCGACGAGGCAGAGAGGCTCGGAGTGTCCAGAGTTGAAACACGTGGCGAACAACAGGTTGTGACACGCTTCGTCTCTGGACACTCCCAGCCTCTCTGCCTC

>c25215_g1_i1

CTGGACATGCCAAAGATGTCCTTTCAATTTGCTGGGATACAAGTGGAAACTGGCAGTCTCAACATCAAAAGTATTGACACTATTTCCTGAAGCAGCAGCCAAATATTTTCCAAATCGAGGCTGGAATCTGACTTGTTTAGTTGCTCCCTTAAACATGCGTGTGCACTTAGATTGGTTGACATTCCACAGTCGGATTTCATCGTTACTATCGCATGATGAAAGGAGATTGAACTTCCTTGGGTGAAAATCCACTGACATTACATGCTCAGTGTGCCCTTCAAGCTTGGATATTGATTCACTTGGTCTGGCTGCATCCCATATCTTCACATTTCTATCAAACGAAGATGTTGCAAACGTTGTTGAATTTGGTCTAAACCGTACATCTGTAATGAGATGAGAATGCTCTTCGAAAGTTTTAACAACATCAAAAGTTTCCATGTTCCAAATCAGAACCTTTTTTTCATGCCCTGCACTTGCTAACAACGTCCCATTTGATGAAAAATGGCAGCATATAACCTTAGCCATGCTTGAATGGAGAGAACAAATTTCTTCGAACTTGAAGCCCCTTTCGTGCTTTTTTTTCCCACCACGACGCTTTGAAGCCTTGAATGGAGTGCTTGTACCACCAGCAGCACTGTCATCCTCATAGGACAAGAAAGACTCGGTATTTTCTTCCACAGGGGCATCTTCATTAGCACTCCTATTTTCCAAATGACTGTCTCCAGCTCCTGATGCTGTCCTCCCAGCTCCTGATTCTGTCCTCCTCTCTCTGTCACCCTCTAGTAACTGCTGTTGATCAGGTTGTTGCTGGGGCTCTGCAATTTGTCTCAAGAGGCAACCCACCATCTGATCACTCAAAGGTGGATGCTGATGTTCTGTTGTTGATGTCTCAATCAGTTGTTGGTGGTTTGGAGCCTGCAAAGCTGCGCTTACTACCTGGAGAGGCTCAAGCCATGGGAAATTTGATCAACTTCAGAGGAAAGTACTAAGAAAATTGAATCAAACAGTTATA

>c23362_g1_i1

CACAAATTAATTTAAATTGATAATGAACTTTGGATAACAAGAATTCCACACCAGCCAAACTGGCCAATACCCTATGCAAATTGGGATAAACCCATATGTCTGTAGTTTGTAACCAAGAAGAAAAGAAAAGAAAAGATGAGAAACAAGAAAAAGAGAGAAATAAACAAGATCACAGATAATTCACTCCTAGTCGCTCTCTGATCTCCTACACAGTTTGTAGTCTTCAACGCCTTTGTTTTTTTACAGGGAGGGAACTGAGGAGCTTGCTAAAGTTGAACCGAGTACATCATCATATAGGCTTGCCCCATCCCGACTCTTCTTGTTATCAGCTCTTGGGCTCCCTGAGCTACTGTTTGAAAGTGAACAGAATGTTGACTTTTGCAGGACACCGGTTGGGGAAGACTCTAACTGGCTGCTGCCATCCCAGCCTTCGTTCAAGAGGTTAATAGGTGATGTGTTCGACGATGATGAGTTCTTGGAGTTCACACAGCTACTGGTGTTGGTCAGGACTTCCCCTAAGGGACCCCCCATCGAACTTCTCCAAGAGATAGGTACCCAATTTGTTTGCTGATCTGTTGGTTCGCTTTGGTCATTGCTCACCCCCAAATTTTGTGTTGGCTCGAACTCACGAGAGAGCCTCAGCGGTGAGAGTCCAAGCTTGTCTTGTGTGGGTGAGGATGAGGATGAGGAGAAGTCCGACGCCATAGGGATTGCCATTGAGAGCTGAGTCCAGTCTGATTTTAAATCCTCAGGCCAAGTAACAACTGATTGACTGGACTGGTCCTTGGGCCAATCATCGATGAACTGGTGAAGCAGATTTTGGTCTTGTGTTTCCTGATCAGTGAAATCAAGGAAAGAACCACCAAAATCTTTGGGAATGTAAGAGCTCCTATGTGAAGGGTTGAGAAGTGAATCAGTAGAAACAAGACCAAACTCTGATAGCGACGATTCTTCCATAGGGAAATCTCGTTTCGGGATGTTAAAACTCGAAGCATTAGATTCAGGCTTGATGCTAGAAGCAGACATCACGGAGAGACCCTGTGGATCTTGCATTCTGTTGGCAAGGGCATCAACAGAAGAATTGGCGGCAGCGGCATCAGCAGGAGGAGGCTCCGAGCTTTTGAACTGCGGCTGGGATGCACCACTGACCGACGTTGGCATTGATGAGGTTACCGGAGGCGCCACTTCCTTTGAATTTGATCTCCTAGAGGCACCATGGCCTGTCTGGCCTTCCACAGGCTTTCTTGAACGATGGCGGCCCCTGTTAATGTGCCTCTCGCAGTACTTCTGGTCTGCTACAGCATCTCTTGAGCAACGCCACTTCTTCCCATCCGTTCGACGACACCTCCCAGGTTCAGGATCAGCATTGCCGGAATATCCCAGATGGAAAGACCCCCAACCTAATGAGTTGGGAGAGGGGCCAGACAAGCCATAAGGGTAGAGGGCCTTCTTGAGAGGAATGAGCAAATTGGATGGCACAGGCGCATTTGCATTCAGGTACTTGTAGATCAAGGCCTGGTGCTCTAGCTCAATCCACTGTGAAGGAGTAAATGGTCCTTTAGCCGCTGCATAAAGCCCATGCATGTTTGCATTCAAGCTTCCAGACCCATAACCTGCATTTCTAGCATAGGGAGAAGAGGGAGTAGGCATGCGCTGGTAGTATGCAAAATCTGAGCTTGCTCCTCCATCTTTGCTCAGAAAAGTGACCTCTGATTTATTGGAGGCAAAGCTCAGCATTTGCTCTTGTCTGGGAGGAGCCAGAGAACTAGATCTCATCAGGTGCTGAGGAGGAGGCCCCTGAGGCAATGGCATTGTCCTGGGAGACGACATGTCGTCGGGTTTGGAGACCCTCCAGTCATCCTCAACAGGCCATGATCTCTGTTGCTTGAAAGACAACCCAGATCCATGACCCGGTTTGGTCTCGGGTCCTGAGACATGAGAATGAGCTGTTGCTCCTTCTCCGGGTTCAGGACCCATCAGGCAATCCATGTCCATACCACCCACCACCCCAAAATCCATTTTGGACACCAATCTAGAACAGGGTCTCTGAGAAGCAGCATGATGAGTTCATTAGCAGCAAAGAAGAAGAAGAAGTTTCAAATGGTTTGGGGAGAGAGAGAATGAAGAAGCGCATCTTGGGTGGGTTTGTAATACTAGTTTTGGGTTTTGGGAGCTGAATATTTTAAAGGTGTGGTCTTTGAGAAGTTAAAAATAA

>c20259_g1_i1

AAGCAAATACTCGATCAGCTATTAACACACATATAGCCACACGATTCATTAAGGTGCAATTGCAGTAGCTAGCTAGCACACGTACATGACAAGGGTTATCAGTATGTCTATTGTAGCTCAGACACACACCTCAATATTATTACAAGTTCCTCGATCATTACTTAGTCATAATAAGATTAGCTCAACTACTATCTGAGCGCGATAATATTACAAACCATGAAATTTAACTGAACTGATAGAAAGCATTAGTTTCTTGATCGAAAAGAAGACAGGAGCTAGGGCACCATCACTAGCATTACATATAGGCAAGATATATGTTATTAATTAATTTTCATTCTCTATCGTGGTCGTCGTGGAGATTAGGCTGAATAGGCTGGACTCGGAAGAAAGACATCTGGGACATCCTCTGATGATACTCCATGTCCCTCAAGTTCTCTTCAGATTTTACGGTCTGTTTGTGCAGCTCATAATTGAGGCGATCATGCTCCTCCTCCGCAGCTTTGTGCTGAGTAGTCGTACGTGTTCCATCCAAAAATATATAGGAAAGTTATTAACCAATGGAGCAAATTAACTACATGATAGCATATGCATTACATATACATTTGGAACATTGAAAATTTAAAGTAAATCAAATAAGTCAATTAATTAAGGATTCCAATAACTTAATTTGATACGTACACTGTCTCTTTGCATCTGGACGACATCTGACTTCTTGCCTCTGACACTAGTAAGGCCATTCTCAAGTGCTTCCTCCAAGTCCCCCAGCTCGCTGTGGTTCAAAGATGTTATGTCCTCCCCCTTCAAATGCCTGAGCTGGACTTGCATGCCGTCATTTTCTTTCTTGATTCTATCCAGTTCATTGG

>c20259_g2_i1

TTTTTTTTTTTTTTTTTTTTGAATTAATGGCTTCAGTACGTATTATTACACACTTGCACAAATAACAGTCATTAAGATGTCCATGAATCCGTTCAGGCACACCTTATTAGTTATTACAAGTTCCACAAATATTATTAGTTAGTCATCAGCAGACTTATTAAGTACTATAATATTACAAACCATGGAAGCATCAATTAATTTCTGGCCAGAAGGACGAGAGATCGAGCTCACATTAATACCAACAGAGAGATGTATATGCATCAATATTACATTCTGTCGTGGAGATTTGGCTGATTAGGCTGGACACGTAAGGCAAAAGGTATCTGGTTGTTGTTGTTGTAGTTCATGTCCCTCAAATTCTCTTCATATTTCATCATTTTGTGCAGCTGGTATGCGAGGCGCTTATTCTCGTCCTCCAGAGCTCTGTTATTTTCTCTAACCGCGTCCATGTACTTCGACATTCTGTCTCTGATACTTGCAAGGCCATTTTCAATTGCTTCCTCTAAGGCCATCAGGTCCACATGGTTCAGAGATGTTATGTCTTCCCCCTTCAAATGCCGAAGCTCAATTTGCATGCCATCATTGTCTTTCTTGACTCTATCCACTTCATTGCAGAGGTTCTGTGAGATTTTGTATATATTTCTTTCAGTATTAGTTCATGTATAATAATTTATCAGAAACTAAAAAGAAAGAAAACTAATTAACCAAAATCGTGATACGATCGATCGATAGTATATTAATAGGATTACTATTAATAGAAATACAGATTAATAACCCAATAAGGGTCTATATGCACACGCAGATCGATCCGTTAATAGAAAAATGTATAGACCGATAATATCAATGCCTGAAGTAGCTAGCAGTCAAGGAACCAACAAGAAAAGACGTTCATTAATTATTATATATTTGGGGTGCTTATAAAGATCTATCAACGAAAATTCTATCAATTTTGTAGCATAGAAAAGCAAGGGAGAGAAACAACCAGATCAGTTCATAGATCGATGTTACATTTCATCTGTCTACAGTTCCATTTTTTTTTTTCTCCATAGACATACAAAACTATCAGAGAGCTTAACAAGAAAGAGATCAAACCAAGGAGCAGTACTGCAGATATCCTATTTGTAGATCTGTTCACACAAATCCCACTTCATGTTTCTTATCATAAATTTAGTATAAAATGAAAAATAAAATAAAATAAAGAGAGAGAACCAAAACCTCATGCTTGGCATCCCATAACCTCTTTCCAGACTGTGAGTGGTATTTGTCCAAGATTTTCATCCGCGTTTCCTGAGGGCCGCTGCAGTATTCAACCATTTTTCCAGAGCTAGCAATGATAATAAGAGAAACCTTAGCATCACAAAGAACAGTGATTTCCTTAGCCTTCTTGATGATCCCATTCTTTCTCTTAGAATAGGTCACCTGCCTGTTGCTTGAGTTTTCAATCCTCTTAATCTCAATCTTACCCCTCCCCATCTTTCACCACAGTATCTTTCTTATTCTTCTCTCTGTATCTGAACTCTTTCTTCTTGCTCTATTCCGTGAAGAAAGAGAAGCTAGTTCTCTTAGAGATCAGGAGAGATAATGTAAAAAGCAGAAGGCTCAAAGTAATTAAGACATATGTTGGACCATTTTCT

>c21688_g1_i1

CTTCTCTCCCCCTCGTCTTCTTTAAATATAAGCTCTCTGCAGTCTGGCTCCTCTTCACAAACCATCTCTGAGTCTCTCCTGTTTGTAAAACTCAGAACCCTAAACCATGTTGGCCATGCTGGTGCCTGCTCCTGCTGAGCAAACCCCTATGGTTTTCGACTCCCAAGTCATTCAGTACCAATCGAACATCCCTTCCCAGTTCATATGGCCGGACCACGAAAAGCCGAGCCCCGACCGGCCGGACCTTCTCGCTCCGGCCATCGACCTCAACGGCTTCCTCAGCGGCGATCCTGCCATCGTCGCGAACTCGATTCGTTCGGTCGACGAGGCCTGCAAGAAGCATGGATTCTTCCTCATTGTGAACCACGGAGTTGATCCGGAGCTCATTGCGAAAGCTCACGAGTACATGGACGTGTTCTTTGAGATGAAGCTCTCGGAGAAGCAAAGGGCTCAGAGGAAGGTTGGTGAGAGCAACGGTTATACTAGTAGCTTCACCGGCAGGTTTAACTCCAAACTTCCATGGAAAGAAACACTTTCTTTCAGGTACTCTGCTGAGAGCCAGAGCTCTACAGTCGTTAAGGACTACTTTGTGAATGTGTTGGGTGAAGATTTCAGAGAAATCGGGAAGCTCTACCAAGAATACTCGGAAGTGATGAACAAACTATCCAATGGGATACTGGAGCTACTGGGACTGAGCCTGGGAGTTGCGCGGGAGTACTTCAAAGAGTTCTACGATGGAAACGATTCGATCATGAGGTTGAACTACTATCCAATGTGCCAAAAACCAGATCTGACACTGGGAACTGGGCCTCACTGTGATCCCACTTCCTTGACAATCCTTCATCAGGATCAAGTAGGGGGACTTGAAGTGTTTTTGGATGAGAAGTGGCACACCGTTACTCCTGTCCTTGGTTCTTTCGTTGTCAACATTGGTGATACTTTCATGGCTCTGTGCAATGGTATTTACAAGAGCTGCTTGCACAGGGCTGTGGTAAACAACAGAACTGTAAGGAAATCTCTTGCTTTCTTTCTCAATCCAAACAAGGACAGAGTAGTGACTCCACCAGCAAGTTTGATCAGTGCTGATAACCCGAGATTGTACCCAGACTTTACTTGGCCTACTCTGCTAGAGTTTACCCAGAAACACTACAGGGCTGACATGAGCACCATTGATGCTTTCTCGGACTGGATTAATGAGCAGAAAGGTGGAGAGGAAAAGAAAGAACAAGGAAACTAGGGAGTCTGTTAAGTGCTTTGGTAGTGTACAAGGGAATAAAGAAATAAAGGGACTCCAGATTATCTAGCTTATTGTTCTAAAGTATAGTTTTCTGTCAATTCAAGCATGAAATGTGGTGGACAAGGCTTGGGCCTATGGTCCCGACCAGATGAGCCCCAAAACTGGGGAATTGGTATTTTAAACCTCATTTTAGGAAATGCTAATGGCAAACTCTGGGGTACTTTTCACATTTCAGGGCTGAAATATCAAATGATAATATCAAGGATAACAAACATAATATCAAAGGAAC

>c20554_g1_i1

CACCAGTTTCCCACACACATTCATTTCTTTCTGGGGTGTTCAAAATCTAAAACCATGGACTCTCTTATCCAACAAGCCTTACAACAACCCGCTCCTATTGCGCTCCTTTCCATGATCCCTGTTCTATTTCTCCTCGGCGTATTTTTCAGGTCCCGACGGCGCTTACCTTATCCGCCGGGACCTAAGGGTCTGCCCATCATCGGTAACATGCTGATGATGGACCAGATAACCCACCGTGGCCTGGCCAAACTAGCCAAACAGTACGGCGGCATCTTCCACCTCCGCATGGGGTTCTTGCACATGGTGGCTGTCTCGACCGCTGACGTCGCTCGGCAGGTTCTTCAAGTACAAGACAACATTTTCTCGAACCGACCGGCGACCATCGCCATCAGCTACCTAACCTACGACCGGGCCGACATGGCCTTCGCTCACTATGGACCCTTTTGGCGTCAGATGCGTAAGCTGTGCGTGATGAAACTCTTCAGCCGCAAAAGAGCCGAGTCGTGGGAGTCCGTGAGGGACGAGGTCGACTCGGCCGTGAGGACCGTGGCGGCCAACACCGGCTCGGCCGTCAACATCGGGGAGCTGGTGTTTTCGCTCACCAAGAATATTATTTACAGGGCTGCGTTTGGGACCAGCTCGAATGAAGGGCAGGACGAGTTCATTGGCATACTGCAGGAATTTTCCAAGTTGTTTGGGGCATTCAATATTGCGGATTTCATTCCCTGTCTCGGGTGGGTAGACCCGCAAGGGCTTAACAACAGGCTGGCTAAGGCTCGTGGGTCGCTGGACAAGTTCATCGACACCATCATAGACGACCACTTGCAGAAGAAGAAAAAGACGGGGAGTTTGGATGAAGGTGAAACTGACATGGTGGATGACTTGTTAGCGTTTTACAGTGAAGAGGCAAAAGTGAATGAGTCGGAGGATAATTTACAAAACGCCATCAAACTTACCAGAGATAACATCAAAGCCATCATCATGGACGTGATGTTTGGCGGGACAGAGACGGTGGCGTCGGCGATAGAGTGGGCCATGTCGGAGCTAATGAGAAGTCCAGAAGACCTAAAGAAGGTCCAACAGGAGCTCGCTGATGTTGTGGGCCTAGAGCGCAGGATCGAAGAGAGCGACTTCGAGAAGCTGACTTACCTAAAATGCGCACTCAAAGAGACACTCCGGCTGCACCCGCCGATCCCTCTCCTCCTTCACGAGACGTCGGAGGACGCGGAGGTCGCCGGCTACAGCATCCCTAAGAAGTCACGCGTGATGATCAACGCGTGGGCGATTGGGCGTGACAAGGACTCATGGGAAGACGCCGAGAGTTTCAAGCCCTCGAGGTTTCTCAAGGAAGGAGTTCCGGACTTCAAGGGGAGTAACTTCGAGTTCATTCCGTTCGGGTCGGGTCGGAGGTCGTGCCCAGGGATGCAGCTGGGGCTGTACGCGCTGGAGCTGGCGGTGGGCCACTTGCTTCATTGTTTCAGTTGGGAGTTGCCTGATGGGATGAAACCTAGTGAGCTTGAAATGAACGACGTGTTTGGACTCACGGCGCCGAGAGCAAGTCGACTCGTCGCCGTACCGACAAAAAGGGTCGTGTGTCCACTCTGAGGAAGAGTCTGAGTAATGAGTTCAATGTCAAGTATACATACCACCAAGACGTCGGAAAAGGTAAATTGAATGGTAATTTGGGAAGAACCCAGAAAAAGAAATAATGTAATAGGGACAACAGGAAAAAGAGGGGATTTGAATTCTCTTCCATTGTGTATAGACACTCGTTTTCATTTGTTTCTTGTGGAAAATTGTCTTGGATTTTCATCTATTTTTGTTCTTTTTTCCTTACTTTTGCTATTGTATTCAACAAAATGACAAATCAATATCTCTTTATTTGAC

>c18395_g2_i1

CTTTCCATGCAATTATTAGCCAGCAGCTGGGACATAATTAATAGCAAGGACCAGGCCCCTAGCTATCTCATGTTTCCTATTCTCTCTTTTCTCAATCTCAAGAATTTGGAGGAATAACATTAGAACCAATAATTGGAGAAGAAGGAAAAAAAAAAAAGTGTATATTACCCTCATCTCTTTTCTCAGTCTCAAACACTCGGAACCAACACGACTCTTGTTGTGGTTTTTCTCTCTGAAAATGAGCCAGTGTGTTCCCAGCTGGGATCTTGATGACAGTAGTCCACCGCCGAGGCTCTCTCTTCGTTCTCACTCCAATTCCACCGCTCCTGATGTTCCCATGTTCGACTATGAAGTAGCAGAGCTGACATGGGAAAATGGCCAGGTGGCCATGCACGGCTTGGGTCTACCGCGCTTGCCTTCCAAACCTGCATCCTGGGACAAGCCACGCGCTACTGGTACTCTGGAGTCCATAGTCAACCAAGCCACCTGTACTTTGCCTCGGTTTTCCAAGCCTCCCTTTGATAACACCATCACTTCTGCCACTGGCGGTGGTGCTAACAAAGTAGTGACCTGGTTCGATCATCACTGTCGTGTTGCCACCTCCGGTGCTCCCTCCAACACCATGACCATGGACGCGTTGGTCCCTTGCAGGAACAACCAACCTGACGAACCAAACAGTAGCCTTATGATAGACTCTGTAGATACCGTGCCAGGAGTGCTCGGCAGTTCTGGCACGGGCGTGATCAATGGGTGCTCCACTCGAGTGGGGTCGTGCAGCGCTACCGCGACCCACAACGAGGACACCCGCCGACTGGGAAAACACAAGGCGGGCGTTGCACGTGTACCTGCTACACCCGAGTGCAGATCCCCCAGCGTGAGCGGTAGTGCCACGTTTGGGATGGATAGCCAGCAAGCCACACTTGATACTAAGGGAAGCGACCGTGACTCAGTTTGTCACAGCAGACCACAGAGAGAGGAAAGCGACGAGGAGGAGAGAAAGAAGCGAAGTACTGGAAAATCCTCAGTTTCTACAAAAAGGAGTAGGGCTGCTGCTATACATAACCAATCTGAACGTAAAAGGAGAGATAAGATCAACCAGAGGATGAAGACGCTGCAGAAGCTGGTCCCAAATTCCAGTAAGACTGATAAAGCTTCAATGCTGGACGAAGTGATTGAATACTTGAAACAATTGCAAGCTCAAGTCCATGTGATAAGCAGAATGAACATGCCAGCCATGATGTTGCCTATGGCTATGCAGCAACAACTTCAAATGTCTATGATGGCCCCAAGAAATATGGGCATGGGGATTGGGATGGGAATGGGAATGGGAATGGG

>c18389_g2_i1

CCACATTATCCCACTATATATGATCGCGGTATCCTTGCCAAGAAATACACCTAAACATAGACTAAAACCAACACTTGGACTGAGTGAAGATGATTTTCCCTATATTTTTCACATTTGTTCTCCTCCTTTCCATTTCCGATGCTGCAGTGCAGGACTTCTGTGTTGCAGACTACAAGGCTCCAGAAGGCCCTGCAGGCTACTCCTGCAAGAAGGCTGCAAAAGTTACCGTAGATGACTTTGTATTCTCTGGCCTAGGTGTTGCTGGTAACACCACAAACATTATCAAAGCTGCAGTCACCCCAGCATTTGCTGCTCAATTTCCTGGTGTTAACGGCCTTGGCATTTCGCTGGCTCGTCTAGACCTGGCTCCTGGTGGAGTTATCCCATTTCACACACACCCTGGAGCTTCAGAAGTCTTGATTGTTACGCAAGGAACCATAATTGCCGGGTTCATTTCATCAGCTAACACAGTTTATCTGAAAACCCTTAAGATGGGAGATCTTATGGTGTTCCCTCAAGGGTTATTGCACTTTCAAGTGAATGGAGGTAATACTTCAGCAATTGCCTTCCCTAGCTTCAGCAGTCCAAGCCCCGGTCTGCAGATTCTGGACTTTGCACTTTTCCAAAACGATTTGCCTACATCATTGATAGCAATGACTACTTTCCTCGATGTTGCTCAGATCAAGAAACTTAAGGGTGTTCTAGGTGGTACTAATTAATTTAGGTTGTGTTGAGATATGTACCAGTGTGTAACTCATTTCCTTTTGTTTCTTTTTGTGATTGAGAAGGCCTCTAAGTGTAGGATTATTCAGGTCAGTAGTGGTTTCTGCTCTGCACTAAATTTATGTATGCTTGTGTAAGTCTTATCGTGTGCTCCAGTTATTGGACCGGTGCATGAGGCAACAATTATGTTGAAGGAAAATGCCACATTTGCATGTTAGTTAACCTGAAATCTCAAATTTCATGTTCTACGTATCTCAGTAGTCACTATCTG
